# Emotional Daily Life Library (E-DLL): Validation of a database of 3D objects for emotion elicitation

**DOI:** 10.1016/j.ijchp.2026.100690

**Published:** 2026-05-14

**Authors:** Diogo Branco, Mariana Castro Fernandes, Sergi Bermúdez i Badia, Ana Lúcia Faria

**Affiliations:** a Faculty of Exact Sciences And Engineering, University of Madeira, Funchal, Portugal; b NOVA Laboratory for Computer Science and Informatics, Lisbon, Portugal; c Faculty of Arts and Humanities, University of Madeira, Funchal, Portugal; d ARDITI - Agência Regional para o Desenvolvimento da Investigação, Tecnologia e Inovação, Funchal, Portugal

**Keywords:** E-DLL, SEEDs, 3D Objects, Emotion elicitation

## Abstract

Research on emotional perception often relies on 2D stimuli or highly affective images, limiting ecological validity. We introduce the Emotional Daily Life Library (E-DLL), a database of 132 rotating 3D everyday objects with comprehensive perceptual, cognitive, and emotional normative ratings. In 52 adults, participants provided dimensional (valence–arousal–dominance) and categorical emotion ratings, alongside assessments of recognition, naming, familiarity, contact, usage, and visual complexity. Personality traits (NEO-FFI) and depressive symptoms (BDI-II) were measured to examine individual differences.

Cumulative Link Mixed Models (CLMMs) revealed that valence ratings were negatively influenced by the interaction of Neuroticism and subclinical depressive symptoms. For arousal, higher Neuroticism and Conscientiousness demonstrated marginal positive associations, while dominance ratings were unaffected. Generalized Linear Mixed Models (GLMMs) for categorical labels indicated that while emotional attributions were primarily driven by stimulus properties, traits such as Neuroticism and Extraversion significantly predicted the perception of negative emotions (e.g., Sadness). Spearman correlations identified interrelationships among cognitive and perceptual dimensions, and redundant variables (Contact and Usage) were combined into a composite Object Interaction score.

By integrating neutral, immersive 3D stimuli with rich multidimensional annotations, E-DLL provides a controlled and ecologically valid tool for experimental and clinical research. Its applicability includes cognitive training, VR-based interventions, and personalized neurorehabilitation platforms such as NeuroAIreh@b, supporting investigations of affective biases and optimizing daily life–oriented therapeutic interventions.

## Highlights

• Introduces E-DLL: 132 everyday 3D objects with cognitive & emotional ratings.
• Neutral, low-arousal objects provide ecologically valid stimuli for clinical research.
• Personality traits & subclinical depression symptoms modulate neutral object ratings.
• Composite metrics and cognitive dimensions enhance object-level characterization.
• Supports clinical applications like VR training and personalized interventions.

## Introduction

Emotions have traditionally been conceptualized through two major theoretical approaches. Discrete models classify emotions into fundamental categories such as happiness, fear, or disgust (Ekman, 1999), whereas dimensional models characterize affective experience along continuous axes, most commonly valence, arousal, and dominance (Bradley & Lang, 1994). Empirical investigations of emotion frequently rely on Standardized Emotion Elicitation Databases (SEEDs), which provide validated stimuli capable of reliably inducing emotional states under controlled laboratory conditions. SEEDs exist across multiple modalities, including audio (Hill and Palmer, 2010, Wu et al., 2024), video (Baraly et al., 2020, Carvalho et al., 2012, Melhart et al., 2022), images (Carretié et al., 2019, Lang et al., 2008, Prantner et al., 2024), and multimodal materials (Barthet et al., 2024, Song et al., 2019, Xue et al., 2022). A comprehensive index of these resources can be found in the KAPODI database (Diconne et al., 2022).

Although SEEDs permit precise manipulation of affective states (Horvat et al., 2014), they only partially approximate real-life emotional experiences. Daily situations are inherently dynamic, multisensory, and interactive, features that are difficult to reproduce using static images or linear audiovisual stimuli. For instance, the International Affective Picture System (IAPS) includes 1182 validated colored images (Branco et al., 2023, Lang et al., 2008), but its static nature limits ecological validity. Video stimuli typically evoke stronger and more sustained emotional responses due to their dynamic and narrative properties (Rottenberg et al., 2007). Nevertheless, both images and videos lack interaction, which is a fundamental component of real-world emotional experience.

Virtual Reality (VR) offers a pathway toward higher ecological validity by enabling immersive and interactive environments. VR systems, including head-mounted displays (HMDs) and Cave Automatic Virtual Environment (CAVE) systems, provide multisensory stimulation and dynamic visual updates based on user movement (Cruz-Neira et al., 1993, Gonçalves et al., 2021). As VR technology has become more affordable and widely accessible, its use in assessment, intervention, and neurorehabilitation has expanded substantially, often demonstrating effectiveness comparable to or exceeding that of traditional approaches (Cameirão et al., 2010, Faria et al., 2016, Paulino et al., 2019, Silva et al., 2021).

Within VR-based research, the use of 3D scenarios has been particularly valuable for simulating everyday activities with high ecological validity (Faria et al., 2023, Parsons, 2015). However, constructing such environments requires access to validated 3D objects with well-characterized perceptual and cognitive properties. Object recognizability, naming agreement, familiarity, prior contact, usage frequency, and visual complexity influence processing demands and determine the appropriateness of stimuli for specific populations. Several 3D object databases have addressed these aspects, although they generally do not incorporate emotional characteristics.

Peeters (2018) validated 147 real-scale 3D objects presented in a CAVE environment, collecting ratings on name agreement, image agreement, familiarity, visual complexity, and lexical attributes. Popic et al. (2020) collected online ratings for 121 rotating grayscale objects, measuring name agreement, familiarity, and visual complexity. Tromp et al. (2020) introduced the OpenVirtualObjects (OVO) dataset of 124 items, which younger and older adults rated on recognizability, familiarity, visual detail, physical contact, and frequency of use, alongside naming and categorization tasks. Although these datasets provide valuable perceptual and cognitive validation, they do not include affective characterization.

An exception is the Base de Dados de Imagens Afectivas 3D (BDIA3D) (Monteiro et al., 2011), which comprises 131 objects validated on valence and arousal using the Self-Assessment Manikin (SAM). These objects were subsequently used to construct the 3D Affective Inducing Scenarios (3DAIS), which included pleasant, unpleasant, and neutral environments and were validated through both self-report and functional magnetic resonance imaging (fMRI) (Dores et al., 2013). Although these studies provide important contributions, BDIA3D does not integrate emotional evaluations with perceptual and cognitive dimensions, limiting the capacity to examine interactions between emotion, cognition, and perception.

In the present study, we introduce the Emotional Daily Life Library (E-DLL), a database of 132 3D everyday objects that integrates perceptual, cognitive, and emotional validation within a single framework. While the E-DLL was explicitly designed to supply assets for immersive VR applications, the present normative validation was conducted using continuous rotating 2D videos presented on standard computer monitors. Consequently, the ecological relevance of the database in the current study derives from the highly realistic, everyday nature of the 3D objects themselves, rather than from an immersive testing environment. Methodological continuity with prior 3D object databases (Peeters, 2018, Popic et al., 2020, Tromp et al., 2020) is maintained through the assessment of recognizability, naming agreement, familiarity, prior contact, usage frequency, and visual complexity. Crucially, the E-DLL extends previous work by incorporating dual emotional characterization: (1) categorical ratings based on Ekman’s basic emotion framework (Ekman, 1999), and (2) dimensional ratings using the full valence–arousal–dominance structure of the SAM (Bradley & Lang, 1994), thereby surpassing earlier efforts restricted to valence and arousal.

The study further examines interindividual differences in emotional evaluation by incorporating the Beck Depression Inventory-II (BDI-II) (Beck et al., 1996, Campos and Gonçalves, 2011) and the NEO Five-Factor Inventory (NEO-FFI) (Costa and McCrae, 1989, Magalhães et al., 2014). These measures allow us to quantify how depressive symptoms and personality traits modulate emotional responses to the objects, providing an analytical dimension that is largely absent from prior SEEDs and 3D object validation studies.

A central strength of the E-DLL is the clinically informed selection of objects. All items were selected by experienced clinicians for their anticipated use in daily life activities. Neutral stimuli are essential in clinical and neurorehabilitation research because they provide stable baselines and minimize unintended emotional activation. This is particularly relevant given that emotional valence and arousal systematically influence memory, attention, and general cognitive performance (Jefferies et al., 2008, Pereira et al., 2023, Schweizer et al., 2019), and that validated emotional properties, including neutrality, can modulate therapeutic outcomes such as pain perception (Shaygan et al., 2017). Moreover, 3D stimuli elicit stronger emotional and physiological engagement than 2D counterparts (Tian et al., 2021), making validated neutral 3D objects especially suitable for VR-based interventions.

By combining cognitive, perceptual, and emotional characterization — and by quantifying the influence of depressive symptoms and personality traits — the E-DLL provides a comprehensive and ecologically valid resource for research in affective science, cognitive psychology, neuroscience, and virtual rehabilitation. The E-DLL has already been applied in clinical settings, including its integration into the NeuroAIreh@b platform, which employs artificial intelligence to personalize neurorehabilitation based on individual cognitive profiles through tasks that simulate daily life activities (Câmara et al., 2025, Câmara et al., 2024, Faria et al., 2024, Paulino et al., 2022).

## Methodology

### Participants

A total of 52 Portuguese participants (34 females, 18 males) with a mean age of 26.06 years (± 7.38) were recruited for the study. All participants were Portuguese nationals, primarily university students. Informed consent was obtained from all participants, and no monetary compensation was provided.

The inclusion criteria specified participants to be Portuguese nationals aged 18 years or older, able to read and write. The primary aim of this study was to establish normative validation of the database within a healthy, non-clinical population. Therefore, to ensure clinical depressive symptoms did not bias the emotional responses, participants who scored above 13 on the Beck Depression Inventory (BDI-II) (Beck et al., 1996), indicating moderate to severe depressive symptoms, were excluded. This exclusion was crucial for maintaining the validity of emotional stimuli assessment under psychologically neutral conditions. However, within this healthy normative sample, we also aimed to evaluate how natural, non-clinical individual differences influence emotional appraisal. To capture these stable traits, participants were assessed using the NEO Five-Factor Inventory (NEO-FFI) (Costa and McCrae, 1989, Magalhães et al., 2014). Because core personality traits, such as Neuroticism, fundamentally interact with current subclinical mood states to modulate emotional processing (Kotov et al., 2010, Rusting, 1998), we retained the subclinical variance in BDI-II scores (< 13). This approach allowed us to explicitly model the interaction between trait Neuroticism and subclinical negative affect, providing a comprehensive evaluation of the affective ratings without compromising the non-clinical foundation of the sample. Initially, 73 participants were recruited; however, 2 were excluded due to technical issues with the monitor, and 19 were excluded based on high BDI-II scores. Ethical approval for the study was granted by the University of Madeira ethics board with the reference number P106.

### Materials

**Fig. 1:**
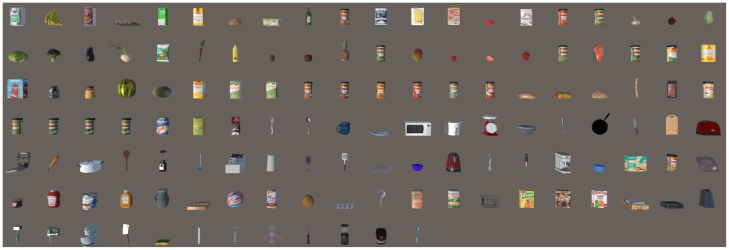
The 132 3D Objects included on the E-DLL.

A total of 132 3D objects (Fig. 1) were used. These assets were used in previous studies that simulated daily life activities for neurorehabilitation (Faria et al., 2024, Paulino et al., 2022). 18 of these were created by designers in our lab, the other assets were adapted from *Sketchfab* (https://sketchfab.com/). For the optimization process, the asset polygons were brought down to a maximum of 2000 polygons using *Simplygon* (https://www.simplygon.com/). The texture added to the objects’ surfaces was either custom-made or taken from freely available textures from the Internet.

### Instruments

The following psychological assessment instruments were applied:

Beck Depression Inventory (BDI-II): The BDI- II (Beck et al., 1996) comprises 21 items, each addressing a specific symptom of depression (e.g., “1. Sadness” with response options ranging from 0 - “I do not feel sad.” to 3 - “I am so sad or unhappy that I can’t stand it”.). Participants evaluate the severity of their symptoms on a 4-point scale from 0 to 3, with higher scores reflecting more severe depressive symptomatology. For this study, we used the European Portuguese version of the Beck Depression Inventory-II (BDI-II) , which has previously demonstrated excellent internal consistency in normative samples (α=0.90–0.91) (Campos & Gonçalves, 2011). This instrument was utilized as the primary inclusion criterion, establishing a cutoff score of ≤ 13 for participant inclusion.

NEO Five-Factor Inventory (NEO-FFI): We used the European Portuguese version of the NEO-FFI (Costa and McCrae, 1989, Magalhães et al., 2014), a 60-item questionnaire assessing five personality traits:

• Neuroticism: Includes emotional instability and negative emotions. Higher scores indicate more distress.
• Extraversion: Items that assess sociability and enthusiasm. High scores reflect outgoing behavior.
• Openness to Experience: Gauges creativity and curiosity. Higher scores indicate more openness.
• Agreeableness: Reflects compassion and cooperation. High scores show more empathy.
• Conscientiousness: Items that measure organization and reliability. Higher scores indicate more diligence.

The validated Portuguese version has demonstrated good internal consistency with theoretical reliabilities (Cronbach’s α) ranging from 0.71 to 0.81 across the five factors (Magalhães et al., 2014).

### Procedure

A QR code containing a link for registration for the study was sent via e-mail to multiple institutional listings and used for in-person recruitment. Up to 48 h before the study, potential participants were sent an email containing both a sociodemographic questionnaire and the Beck Depression Inventory (BDI-II) (Beck et al., 1996, Campos and Gonçalves, 2011). The experiment consisted of a single session where participants were first asked to fill out the European Portuguese version of the NEO-FFI (Costa and McCrae, 1989, Magalhães et al., 2014). Afterwards, they completed a brief training round in which they rated two objects, an orange and a sweet potato, that were not included in the main database (E-DLL).

**Fig. 2:**
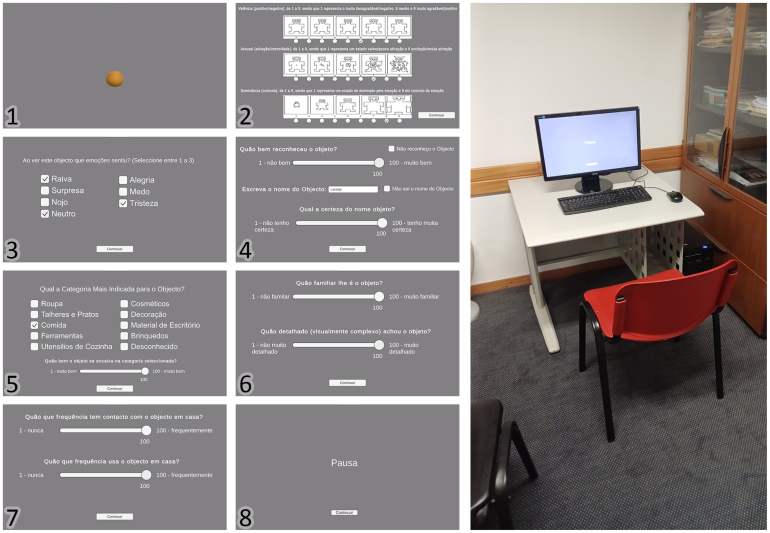
Screens presented to the participant, labeled 1–7 for each object. The Pause screen (8) appears between objects 66 and 67. The setup used is also shown.

The experiment had three phases: (1) Visualization, (2) Emotional Classification of the Object, and (3) Object Classification. This procedure mimics existing studies in the area of 3D object validation (Peeters, 2018, Popic et al., 2020, Tromp et al., 2020) (Fig. 2). Each session lasted approximately 1 h and 20 min. Midway through the session (between objects 66 and 67), participants encountered a pause screen, offering the option to take a short break or continue immediately.

Both the training and main experiment were conducted using custom software developed in Unity (Technologies, 2025), which enabled integrated video presentation and data collection. Data was stored in Comma-Separated Values (CSV) format. The experiments were run on a desktop computer with the following specifications: Windows 10 operating system, Intel Core i5-4440 CPU @ 3.10 GHz, 8 GB DDR3 RAM @ 1600 MHz, and an AMD Radeon R7 200 series GPU (Fig. 2).

The following steps were followed:

1. **Visualization:** The objects were recorded rotating around their central vertical axis at a speed of 60°per second for 8 s, ensuring that all sides were visible. Participants viewed the videos, rendered at a resolution of 1920 × 1080 and 60 FPS, on an *Asus* monitor with the same resolution, with a 55 cm screen size (Fig. 2). The presentation order of the 132 3D objects was randomized for each participant to reduce order effects.
2. **Emotional Classification:** For emotional classification, the participants evaluated the objects using a dimensional assessment of emotion via the Self-Assessment Manikin (SAM) (Bradley & Lang, 1994), which consists of valence (1 - very negative, 9 - very positive), arousal (1 - not excited, 9 - very excited), and dominance (1 - dominated by the emotion, 9 - in control of the emotion) and a categorical assessment by being presented with Ekman’s six basic emotions (Anger, Surprise, Disgust, Joy, Fear, and Sadness) (Ekman, 1999), along with the additional category “Neutral” and having to choose between 1 to 3 emotions.
3. **Object Classification:** During the object classification task, participants interacted with several components to assess their recognition and understanding of each object. For applicable measures, they used a 1 (low) to 100 (high) rating scale.
  • Recognition: Participants first indicated whether they recognized the object. If not, they selected “I don’t recognize the object.” If they did, they rated their level of recognition.
  • Naming: After the recognition rating, participants wrote down the name of the object and rated its certainty. If they did not know the object’s name, they selected the “I do not know the name” option.
  • Category Selection: Participants categorized the object by selecting the most appropriate category from ten options: clothing, cosmetics, cutlery and plates, decoration, food, office supplies, tools, toys, kitchen utensils, or unknown and then rated its certainty.
  • Familiarity: Participants rated how familiar the object was.
  • Visual Detail: Participants rated how visually detailed the 3D Model of the object appeared.
  • Contact and Usage Frequency: Participants rated how often they encountered the object and how frequently they used it at home.

## Data analysis

All data analyses were conducted in R (version 4.5.2).

### Psychological assessments

For the Beck Depression Inventory (BDI) (Beck et al., 1996, Campos and Gonçalves, 2011) and the NEO-FFI (Costa and McCrae, 1989, Magalhães et al., 2014) factors (Neuroticism, Extraversion, Openness to Experience, Agreeableness, and Conscientiousness), we computed descriptive statistics, including mean, standard deviation (SD), median, minimum, maximum, skewness, and kurtosis.

Internal consistency was assessed using the psych package (Revelle, 2025) in the R statistical computing environment. We reported both Cronbach’s alpha (α) and McDonald’s omega total ($\omega_{t}$) to estimate reliability. While Cronbach’s alpha is the traditional metric (Cronbach, 1951), it assumes tau-equivalence (equal factor loadings) across items. Therefore, McDonald’s $\omega_{t}$ was included as a more robust estimator for congeneric models where factor loadings vary (McDonald, 1999). McDonald’s omega was calculated based on a unidimensional factor model using minimum residual factor analysis. Ninety-five percent confidence intervals (95% CI) were reported for Cronbach’s alpha.

### Dimensional ratings - VAD

Each participant rated 132 objects on three affective dimensions: Valence, Arousal, and Dominance (VAD) using a 1–9 integer scale. Descriptive statistics, including the mean, standard deviation (SD), and median, were computed for each object.

### Relationship between personality traits, depressive symptoms, and affective ratings - VAD

All continuous predictor variables, including BDI scores and the five personality factors (Neuroticism, Extraversion, Openness to Experience, Agreeableness, Conscientiousness), were standardized (z-scored) to have a mean of 0 and a standard deviation of 1. Standardization facilitated model convergence and enabled interpretable comparisons of regression coefficients across predictors. As all participants scored below the clinical cutoff for depression (BDI <13), BDI was treated as a continuous measure of *relative depressive symptom severity within a non-clinical range*.

**Data Transformation:** To address estimation instability and non-convergence caused by sparse responses at the extremes of the original 9-point scale, affective ratings were collapsed into a 5-point ordinal scale prior to analysis (i.e., original scores of 1–2 were recoded as 1; 3–4 as 2; 5 as 3; 6–7 as 4; and 8–9 as 5). Sparse category frequencies are known to destabilize estimation in cumulative link models by preventing the reliable calculation of threshold parameters (Agresti, 2010). This transformation mitigated these issues by increasing cell counts while retaining the central neutral category to preserve interpretability. While this approach involves a necessary trade-off between estimation stability and response granularity, it ensured the models could converge reliably.

**Model Specification:** Separate cumulative link mixed models (CLMMs) were fitted for each affective outcome (Valence, Arousal, Dominance) using the clmm function from the ordinal package (Christensen, 2025). Each model included the six standardized predictors (BDI and the Big Five personality traits) as fixed effects. Random intercepts for both *Participant_ID* and *Object_ID* were included as crossed random effects to account for the repeated-measures structure of the data and the shared unmodeled variance inherent to the specific stimuli. To examine potential moderation by depressive symptoms, interaction models were specified including the Neuroticism × BDI interaction, with other personality traits included as covariates.

**Estimation, Convergence and Robustness:** Models were estimated using maximum likelihood with the Hessian evaluated at convergence. To ensure stable estimation, the maximum number of iterations was set to 10,000. In instances where the maximal crossed random-effects structure resulted in non-convergence (as occurred with Dominance), the random effects structure was systematically simplified by removing the stimulus-level intercept to achieve a stable fit. Model stability was explicitly verified by ensuring that the maximum absolute gradient for all reported models was well below the standard 0.01 threshold. Because analytical power calculations are largely unfeasible for complex mixed models with crossed random effects (Brysbaert & Stevens, 2018), and given our modest participant sample size (N=52), a 10,000-iteration Monte Carlo simulation-based sensitivity analysis (via cluster bootstrapping) was conducted to empirically evaluate our design and confirm the stability of the crossed random-effects structure. The simulation utilized the foreach and doRNG packages for reproducible parallel processing (Gaujoux, 2026, Microsoft and Weston, 2022). For each iteration, participants were resampled with replacement to preserve the repeated-measures structure. Crucially, the maximal crossed random-effects models successfully converged in over 97% of these 10,000 bootstrap iterations, further confirming mathematical stability. This empirical sensitivity analysis revealed an estimated probability of 64.09% to detect the critical Neuroticism × BDI interaction for Valence, and 58.46% to detect the main effect of Neuroticism. Furthermore, sensitivity analyses were performed by systematically comparing nested models (main effects vs. interaction models) to confirm that the inclusion of interaction terms did not destabilize the estimates.

**Model Fit and Comparison:** Model fit was evaluated using Akaike Information Criterion (AIC) (Akaike, 2003), Bayesian Information Criterion (BIC) (Schwarz, 1978), and log-likelihood values (Dobson & Barnett, 2018). Likelihood ratio tests were used to formally compare main-effects models against interaction models to determine whether including the Neuroticism × BDI interaction improved model fit.

**Inference:** Fixed-effect estimates are reported as log-odds, odds ratios (OR), and 95% confidence intervals (CI). Statistical significance for fixed effects was evaluated at an α level of .05. Interaction effects were visualized using estimated marginal means (EMMs) across a range of Neuroticism values (−2 to +2 SD) and representative BDI values reflecting *lower* (−1 SD), *average* (0 SD), and *higher* (+1 SD) levels within the observed, non-clinical range, with confidence intervals displayed as shaded ribbons. For interpretation on the latent linear predictor scale, lower predicted values for Valence correspond to a higher probability of low emotional ratings (more negative valence), whereas higher predicted values for Arousal correspond to a higher probability of high emotional ratings (greater arousal). Dominance predictions were interpreted similarly on the latent scale.

### Categorical ratings - Ekman

Each participant provided ratings on seven emotion dimensions: Neutral, Happiness, Fear, Surprise, Anger, Sadness, and Disgust.

For the categorical ratings, we calculated both the *modal emotion agreement* for each individual object and the *global emotion agreement* across all objects. The modal emotion for an object was defined as the emotion (or combination of emotions) selected by the largest number of participants, a procedure adapted from previous studies (Peeters, 2018, Popic et al., 2020, Tromp et al., 2020). A total of 6864 entries (52 participants × 132 objects) were analyzed.

Participants were instructed to select between one to three emotions per object. To ensure consistency, combinations such as “Happiness | Sadness” and “Sadness | Happiness” were treated as identical, as they represent the same emotional response in different orders. The frequency of each emotion and emotion combination was recorded and expressed as a percentage of total responses. These results were subsequently ranked, with a top-10 list of the most frequent emotional responses reported.

### Relationship between personality traits, depressive symptoms, and affective ratings — categorical

Generalized linear mixed models (GLMMs) were used to evaluate the association between BDI scores, Big Five personality traits, and the likelihood of attributing an emotion. All continuous predictor variables, including BDI scores and the five personality factors (Neuroticism, Extraversion, Openness to Experience, Agreeableness, Conscientiousness), were standardized (z-scored) to have a mean of 0 and a standard deviation of 1. Standardization was performed to facilitate model convergence and enable interpretable comparisons of regression coefficients across predictors.

**Model Specification and Estimation:** Separate binary logistic mixed-effects models were fitted for each emotion category using the glmmTMB package (Brooks et al., 2017), which provides robust estimation for binomial data. Each model included the six standardized predictors (BDI and the Big Five personality traits) as fixed effects. To account for the crossed random-effects structure of the task, random intercepts were included for *Participant_ID* (capturing individual differences in response tendencies) and *Object_ID* (capturing stimulus-level variability).

**Sensitivity Analysis and Model Selection:** Given the low baseline endorsement frequencies for certain negative emotions (e.g., Fear, Anger, Sadness), models were systematically evaluated for structural stability, singular fits, and boundary issues. To ensure parsimony and prevent singular fits by over-parametrization (Bolker et al., 2009, Matuschek et al., 2017), a formal data-driven sensitivity analysis was conducted. For each emotion, the full model featuring crossed random effects was compared to a reduced model (retaining only the *Participant_ID* random effect) using Likelihood Ratio Tests (LRTs). If the inclusion of the *Object_ID* random intercept did not significantly improve model fit (p>0.05), the stimulus-level random effect was dropped. Consequently, the final models for Anger and Sadness utilized only the *Participant_ID* random effect, while all other emotions retained the full crossed random-effects structure.

**Inference and Model Fit:** Fixed-effect estimates are reported as odds ratios (OR) with 95% confidence intervals (CI). Model fit was evaluated using Akaike Information Criterion (AIC), Bayesian Information Criterion (BIC), and pseudo-$R^{2}$ statistics. Marginal pseudo-$R^{2}$ represents the proportion of variance explained by fixed effects alone, while conditional pseudo-$R^{2}$ represents the proportion of variance explained by both fixed and random effects (Lüdecke et al., 2021, Nakagawa et al., 2017, Nakagawa and Schielzeth, 2013). Statistical significance for fixed effects was evaluated at an α level of 0.05.

### Object ratings

#### Modal name and name agreement

For this analysis, we closely followed the standardization methodology outlined in Popic et al. (2020), while adapting the analysis framework from Tromp et al. (2020) and Peeters (2018). The **modal name** was defined as the name (or combination of names) provided by the highest number of participants. To ensure that name agreement reflected concept identification rather than lexical preference, individual entries were standardized based on the following criteria adapted from Popic et al. (2020):

1. **Correction of Misspellings** — Minor spelling errors were corrected to maintain consistency.
2. **Removal of Non-Essential Descriptors** — Adjectives that did not alter the core identity of the object were omitted (e.g., “mug” instead of “large mug”).
3. **Normalization of Word Order** — Equivalent composite names with different word structures were treated as the same (e.g., “cutting board” and “board for cutting” or “measuring cup” and “cup for measuring”).
4. **Unification of Synonyms** — A semantic analysis was conducted to merge synonymous responses (e.g., “taça” and “tigela” were considered equivalent). The most frequently used variant was selected as the modal name.

Once the **modal name** was determined, all corresponding responses were recoded to match it. This standardization ensured that minor variations did not impact name agreement, leading to a more accurate representation of naming consistency. To assess naming consistency, we focused exclusively on name agreement (NA). While both Peeters (2018) and Tromp et al. (2020) utilized the H-statistic (Snodgrass & Vanderwart, 1980), a logarithmic measure of lexical diversity, we omitted it from this analysis, consistent with the approach taken by Popic et al. (2020). The H-statistic is highly sensitive to lexical variations (e.g., distinguishing between ”mechanical alarm clock and analog alarm clock”). As noted by Tromp et al. (2020), such strict distinctions can artificially inflate disagreement metrics and “may not be necessary or appropriate for all studies”. Because our standardization and recoding steps explicitly resolve the lexical ambiguity that the H-statistic is designed to measure, we determined that name agreement provided the most accurate representation of the concept. To assess **name agreement**, we calculated the mean and standard deviation for each object, representing the percentage of participants who provided the modal name. Additionally, we recorded the percentage of alternative responses, the proportion of unrecognizable responses (coded as ‘00’), and the percentage of responses labeled as “I Don’t Know the Name” (coded as ‘Unknown’). This provided a comprehensive overview of the objects and their naming patterns.

#### Categorization of the object

Each participant was allowed to select only one category for each object. Following the modal name approach, a modal category for each object was determined by selecting the most frequently chosen category across all participants. For instance, if 50 participants categorized a banana as “Food” (96.15%) and 2 participants categorized it as “Kitchen Utensils” (3.84%), the modal category was defined as “Food”. Categories that received votes but never reached this most frequent status for any object (such as “Unknown” or “Tools”) are therefore represented in the total percentage distribution but not in the final modal category counts. A residual category, stemming from the “I don’t recognize the object” option, was labeled as “Unrecognized.” Additionally, the percentage distribution for each category was calculated to determine the proportional representation of categories across the stimulus set.

#### Objects data analysis

Raw data comprised subjective ratings across seven cognitive and perceptual dimensions: *Recognition, Name Certainty, Familiarity, Category Certainty, Visual Complexity, Contact,* and *Usage*. Prior to aggregation, logical constraints were applied to the raw data. If the “Unrecognizable” or “Unknown” category was selected for an object, the certainty ratings for *Name, Familiarity, Category, Contact,* and *Usage* were automatically set to 0. However, *Visual Complexity* was assessed independently of recognition because the participants were instructed to analyze the rate of detail of 3D objects, therefore, participants’ ratings for visual complexity were retained even if the object was deemed unrecognizable. To derive stable object-level estimates from the participant ratings, a composite score was calculated for each object by computing the row-wise median across all raters. The median was selected as the measure of central tendency to minimize the influence of potential outliers in the rating distributions.

For each aggregated dimension, descriptive statistics (mean, SD, median, range, skewness, and kurtosis) were calculated to characterize the data. Shapiro–Wilk tests indicated significant deviations from normality for most dimensions (p<.05; see Table 6). Accordingly, medians were preferred as the primary measure of central tendency, and non-parametric tests were used for subsequent analyses.

Prior to correlational analyses, a multicollinearity assessment was conducted to ensure the statistical independence of the predictors. Variance Inflation Factor (VIF) analysis revealed severe multicollinearity between the functional dimensions *Contact* (VIF=10.43) and *Usage* (VIF=9.32). This overlap was further confirmed by a near-perfect Spearman rank correlation between *Contact* and *Usage* (ρ=.96, p<.001), indicating that these dimensions captured highly redundant information regarding object interaction. To resolve this issue and prevent Type I error inflation, these two dimensions were averaged into a single composite variable termed **Object Interaction**. Subsequent VIF analysis on the final variable set confirmed that all inflation factors were well below the standard threshold of 5 (VIF range: 1.22–4.80), indicating satisfactory independence.

To examine the interrelationships between dimensions, Spearman’s rank-order correlations (ρ) were computed. To strictly control for the family-wise error rate (Type I error) associated with multiple testing across the 15 unique pairwise comparisons, a Bonferroni correction was applied. Statistical significance was defined as an adjusted p-value <.05. Correlation matrices for the final six dimensions after merging Contact and Usage were visualized using a heatmap. Color intensity represented the strength and direction of Spearman correlations, and statistical significance was annotated with asterisks (* p<.05; ** p<.01; *** p<.001).

### Comparison with other databases

This section compared E-DLL with several 3D object databases, focusing on both emotional ratings and other relevant attributes. For emotional ratings, E-DLL was directly comparable to BDIA 3D (Monteiro et al., 2011) regarding Valence and Arousal dimensions. We reported mean values and standard deviations (SD) for both databases and additionally provided Dominance ratings and categorical emotion labels for E-DLL where applicable. Beyond emotional ratings, we assessed attributes such as Recognition, Name Agreement (NA), Familiarity, Visual Complexity, Contact, and Usage across different databases. For Name Agreement, we restricted quantitative comparisons to databases employing compatible scoring methodologies. Specifically, we computed effect sizes between E-DLL and Popic et al. (2020), as both studies utilized the semantic grouping strategy described in the *Modal Name and Name Agreement* section. We excluded the primary data from Tromp et al. (2020) (OVO) from NA effect size calculations due to the methodological incompatibility previously detailed in Section “Object Ratings”.

For all other attributes, we standardized rating scales to allow for direct comparison. Studies by Peeters (2018) and Popic et al. (2020) utilized 1–5 rating scales, which we converted to a 1–100 scale using the formula:

$$\text{New Value}=1+\frac{\left(\text{Old Value}-1\right)\times \left(100-1\right)}{5-1}$$

After the transformation, we recalculated the mean and SD for each object in these databases. To quantify the magnitude of differences between E-DLL and the other databases, we computed Hedges’ g (Hedges, 1981) for all overlapping attributes where SDs were available. Crucially, the pooled standard deviations used for the Hedges’ g calculations were computed using these newly rescaled 1–100 metrics to ensure mathematical comparability. Hedges’ g provides a standardized measure of effect size, with small, medium, and large thresholds indicating the practical significance of observed differences (Cohen, 2013). Attributes with missing SDs or incompatible methodologies were reported descriptively without effect size calculation.

## Results

### Internal consistency of psychological measures

Descriptive statistics and reliability estimates for all measures are presented in Table 1. Internal consistency was generally good across the measures.

For the BDI, internal consistency estimates were moderate (α=0.67, $\omega_{t}=0.66$). These values are lower than typically reported in clinical samples (α>0.80), likely due to the exclusion of participants scoring above 13, which restricts variance (range restriction) and consequently attenuates reliability coefficients (Cronbach, 1951). The close agreement between α and $\omega_{t}$ suggests that this reflects limited symptom variability rather than structural issues (McDonald, 1999).

For the NEO-FFI factors, reliability was generally high. Neuroticism (α=0.84, $\omega_{t}=0.85$) and Conscientiousness (α=0.84, $\omega_{t}=0.86$) demonstrated the strongest consistency. Extraversion was moderately high (α=0.78, $\omega_{t}=0.79$), while Openness (α=0.70, $\omega_{t}=0.77$) and Agreeableness (α=0.67, $\omega_{t}=0.75$) showed acceptable reliability. As shown in Table 1, the 95% confidence intervals for α indicate that the true reliability for all personality factors likely falls within an acceptable to good range.

**Table 1:** Descriptive statistics and internal consistency for the NEO-FFI personality factors and depressive symptomatology (BDI).

Scale	Items	M±SD	Median	Min	Max	Skew	Kurtosis	α (95% CI)	$\omega_{t}$
BDI-II	21	4.81±3.50	5.00	0.00	12.00	0.28	−1.08	0.67 (0.54–0.80)	0.66
Neuroticism	12	20.65±7.80	22.00	0.00	35.00	−0.38	−0.41	0.84 (0.78–0.91)	0.85
Extraversion	12	30.67±6.55	31.50	15.00	44.00	−0.70	0.27	0.78 (0.69–0.87)	0.79
Openness	12	30.52±6.57	31.00	18.00	42.00	−0.07	−1.00	0.70 (0.59–0.81)	0.77
Agreeableness	12	34.10±5.29	34.00	22.00	45.00	−0.06	−0.66	0.67 (0.55–0.80)	0.75
Conscientiousness	12	35.35±6.70	35.50	13.00	48.00	−0.60	1.11	0.84 (0.77–0.90)	0.86

### Emotional ratings

#### Dimensional ratings (VAD)

**Fig. 3:**
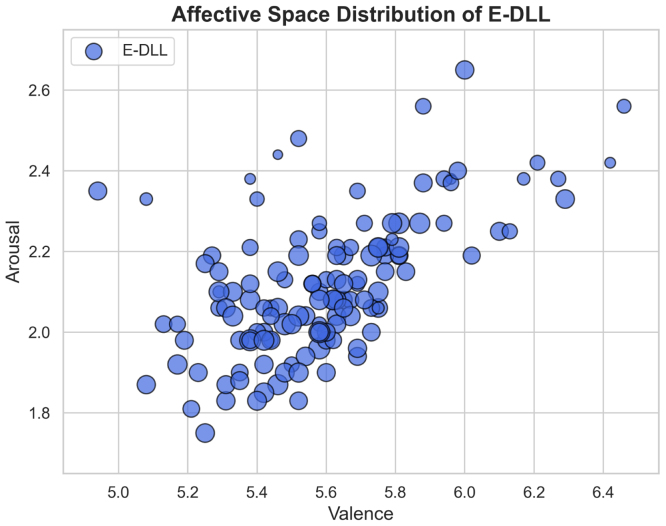
E-DLL items distributed along the affective space, with the mean Valence rating represented on the X-axis and the mean Arousal rating on the Y-axis. The size of the dots represents the mean Dominance rating of each object.

The descriptive statistics indicate neutral valence, low arousal with some variability, and consistently high dominance, providing insight into the emotional characteristics of the sample (see Table 2). In Fig. 3, E-DLL objects are distributed along the affective space, illustrating the relationship between emotional dimensions and control for each item.

**Table 2:** Global descriptive statistics for the emotional dimensions.

Dimension	Mean (SD)	Median	IQR
Valence	5.59 (0.27)	5.00	1.00
Arousal	2.12 (0.17)	1.00	2.00
Dominance	7.64 (0.12)	9.00	3.00

#### Relationship between personality traits, depressive symptoms, and affective ratings - VAD

To account for the ordinal nature of the emotional ratings, the sparsity of extreme responses was addressed by collapsing ratings into a 5-point scale (preserving the neutral midpoint). Cumulative Link Mixed Models (CLMMs) with crossed random effects for both Participant and Object were then fitted to absorb shared stimulus-level variance. Separate models were estimated for Valence, Arousal, and Dominance. Predictors included standardized Big Five personality traits and depressive symptoms (BDI). For each outcome, a main-effects model was compared with an interaction model including the Neuroticism × BDI term (see Table 3). Model stability was confirmed (all maximum gradients <0.01). Additionally, a 10,000-iteration Monte Carlo cluster bootstrap simulation estimated the design’s sensitivity for the Valence interaction at approximately 64%.

**Fig. 4:**
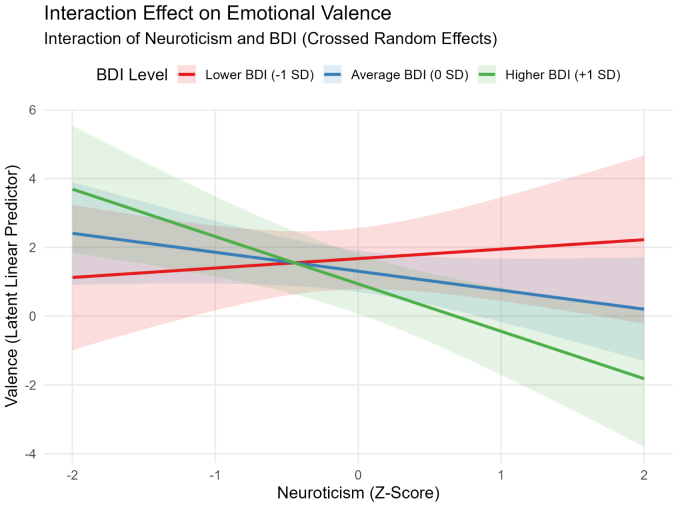
Interaction effect of neuroticism and depressive symptoms (BDI) on emotional valence **Note.** The plot displays predicted latent linear responses from the cumulative link mixed model (CLMM) incorporating crossed random effects. Lower values on the latent linear predictor correspond to more negative emotional valence ratings. The negative association between neuroticism and valence becomes significantly stronger at higher relative BDI scores (p=.015).

**Valence:** In the main-effects model, higher Neuroticism significantly predicted lower valence (OR =0.482, 95% CI [0.237, 0.980], p=.044). The interaction model yielded an improved fit over the main-effects model ($\Delta \chi^{2}\left(1\right)=5.62$, p=.018) and revealed a significant Neuroticism × BDI interaction (OR =0.438, 95% CI [0.225, 0.853], p=.015) (Fig. 4 illustrates this effect).

**Arousal:** Including crossed random effects for both participants and objects absorbed previously unmodeled stimulus variance. A likelihood ratio test confirmed that adding the interaction term did not improve model fit ($\Delta \chi^{2}\left(1\right)=0.01$, p=.906), and the Neuroticism × BDI interaction for Arousal was not significant (OR =1.077, 95% CI [0.316, 3.671], p=.906). In the main-effects model, no predictors reached strict statistical significance, though strong marginal trends emerged indicating that higher Neuroticism (OR =3.153, 95% CI [0.909, 10.935], p=.070) and higher Conscientiousness (OR =3.824, 95% CI [0.982, 14.886], p=.053) were associated with higher emotional arousal ratings.

**Dominance:** Because the crossed random-effects structure failed to converge, a simplified model retaining only the participant-level random intercept was fitted. No significant main or interaction effects emerged for Dominance. The interaction model did not improve fit over the main-effects model ($\Delta \chi^{2}\left(1\right)=1.11$, p=.291), and the Neuroticism × BDI interaction was non-significant (OR =0.407, 95% CI [0.078, 2.122], p=.286), suggesting that dominance ratings were largely independent of personality or depressive symptoms.

**Supplementary Material:** Full table of results, including fixed effects, confidence intervals, AIC, and BIC, for all main and interaction models are provided in Supplementary Material S1.

#### Categorical ratings (Ekman)

The modal emotion across all objects was *Neutral*, with a global agreement of 79.11%. Across 6864 responses (132 3D objects rated by 52 participants), 43 distinct emotional categories were identified, including single and compound emotions. The top 11 emotional responses, along with their frequency and percentage, are summarized in Table 4, suggesting that the 3D objects generally did not provoke strong emotional reactions. Positive emotions such as *Happiness* (5.93%) and *Surprise* (3.10%) were less frequent but notable, indicating some positive engagement. Compound responses, e.g., *Happiness* | *Surprise* (2.77%) and *Happiness* | *Neutral* (1.35%), reflect complex positive emotional reactions.

Negative emotions were rare, with *Disgust* at 2.42%, *Anger* at 0.35%, *Fear* at 0.34%, and *Sadness* at 0.31%. Some negative combinations, such as *Disgust* | *Sadness* (0.29%) and *Disgust* | *Neutral* (1.03%), indicate nuanced emotional reactions to specific objects.

**Table 3:** Neuroticism × BDI Interaction Effects From CLMMs (Crossed Random Effects).

Outcome	OR	95% CI	p	$\Delta \chi^{2}\left(1\right)$	LRT p
Valence	0.438	[0.225, 0.853]	.015	5.62	.018
Arousal	1.077	[0.316, 3.671]	.906	0.01	.906
Dominance	0.407	[0.078, 2.122]	.286	1.11	.291

*Note.* OR = Odds Ratio; CI = 95% confidence interval; BDI = Beck Depression Inventory; $\Delta \chi^{2}\left(1\right)$ = likelihood ratio test (LRT) statistic comparing the main-effects model to the interaction model. The LRT confirms that the Neuroticism × BDI interaction significantly improves overall model fit for Emotional Valence, but not for Arousal or Dominance. Additionally, due to non-convergence of the crossed-effects structure, the estimates for Dominance were derived from a simplified model utilizing only a participant-level random intercept.

Responses involving three or more emotions were infrequent, highlighting the rarity of highly complex emotional experiences. Examples include *Disgust* | *Happiness* | *Surprise* (0.20%) and *Anger* | *Fear* | *Sadness* (0.08%), which occurred in response to emotionally ambiguous or provocative objects.

**Table 4:** Top 11 Most Frequent Emotional Responses Across 132 3D Objects.

Emotion	Frequency	Percentage (%)
Neutral	5430	79.11
Happiness	407	5.93
Surprise	213	3.10
Happiness | Surprise	190	2.77
Disgust	166	2.42
Happiness | Neutral	93	1.35
Disgust | Neutral	71	1.03
Neutral | Surprise	67	0.98
Anger	24	0.35
Fear	23	0.34
Sadness	21	0.31

#### Relationship between personality traits, depressive symptoms, and affective ratings — categorical

The analysis included 6864 observations across 52 participants and 132 stimuli objects. Following a formal sensitivity analysis, random-effect structures were optimized to ensure model stability and prevent singular fits. For Neutral, Happiness, Fear, Surprise, and Disgust, models retained crossed random effects for both participant and object, indicating significant variability at both levels. For Anger and Sadness, likelihood ratio tests indicated that object-level variance was negligible and did not significantly improve model fit (p=.162 and p=.624, respectively); thus, these models were reduced to include only participant-level random effects for parsimony. Across all final models, the intra-class correlation coefficients (ICCs) demonstrated stable clustering, ranging from 0.52 to 0.71, with no boundary issues observed.

**Fixed-Effects Findings:** Generalized linear mixed models (GLMMs) evaluated the association between BDI scores, Big Five personality traits, and the likelihood of attributing specific emotions. A key finding emerged within the low-frequency negative emotions: for Sadness, both Neuroticism (OR=2.63, 95% CI [1.01,6.87], p=.048) and Extraversion (OR=2.36, 95% CI [1.00,5.54], p=.049) were statistically significant positive predictors. Additionally, Fear demonstrated a trending, though non-significant, positive association with Neuroticism (OR=2.24, p=.070). Among the more frequently endorsed emotions, Surprise exhibited a trending negative association with Agreeableness (OR=0.48, 95% CI [0.23,1.01], p=.052). No significant fixed effects were found for Neutral, Happiness, Anger, or Disgust.

**Model Fit and Limitations:** Fixed effects explained a modest proportion of variance (marginal $R^{2}=0.016\text{–}0.150$), while including random effects substantially increased the total variance explained (conditional $R^{2}=0.537\text{–}0.742$). While the total observation count (N=6864) provided robust power for estimating variance components and fixed effects, we acknowledge that the participant-level sample size (N=52) is relatively modest. Though sufficient for stable estimation in mixed-modeling frameworks, this sample size presents a limitation in statistical power, potentially constraining the ability to detect more subtle between-subject individual differences.

**Supplementary Material:** Full results for all predictors and emotions, including odds ratios, confidence intervals, AIC, BIC, and marginal/conditional $R^{2}$, are provided in Supplementary Materials S2 and S3. Furthermore, the complete sensitivity analyses and Likelihood Ratio Tests evaluating the random-effects structures are detailed in Supplementary Material S2b.

### Object identification

#### Recognition

**Fig. 5:**
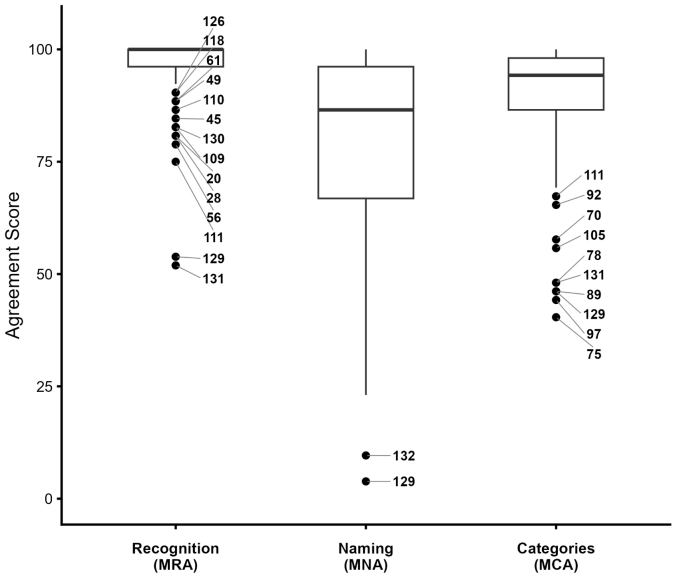
Boxplot showing the distribution of modal recognition agreement (MRA), modal name agreement (MNA), modal category agreement (MCA) across all objects, highlighting outliers with notably lower values.

Participants recognized most objects with a modal recognition agreement (MRA) of 96.50% ± 7.24%. The object with the lowest MRA was ID 131 (“Air Fryer”), with 27 participants marking it as “I Do Not Recognize” (51.92%), followed by object ID 129 (“Peeler”) with 28 participants (53.85%) (Fig. 5). The recognition certainty was 91.83 ± 12.89.

#### Name

The modal name for each object was defined as the name selected by the highest number of participants. The modal name agreement was 83.62% ± 18.68%, indicating the average percentage of participants who agreed on the chosen names. The name certainty was also 83.62 ± 18.68. The objects with the lowest name agreement were ID 129 (“Peeler”), with only 3.84% agreement, and ID 132 (“Spaghetti Spoon”), with 9.61% agreement.

#### Category

The overall Modal Category Agreement (MCA) was 88.91% (± 12.98%). Objects were primarily classified into three modal categories: “Food,” “Kitchen Utensils,” and “Cutlery and Plates.” A residual category, labeled “Unrecognized,” captured instances where participants selected the “I don’t recognize the object” option; this category had minimal impact on the overall analysis. Each of the 132 objects was evaluated by 52 participants, yielding a total of 6864 entries. The mean category certainty was 90.14 (± 8.33). Table 5 summarizes the distribution of objects across categories, detailing the MCA and the proportion of total entries.

**Table 5:** Categorization of Objects: Distribution of Modal Category Agreement (MCA) and Total Entries. Note. “All entries” reflects the total pool of individual categorization responses (N=6864, derived from 52 participants rating 132 objects). Modal Category Agreement (MCA) represents the final classification for the 132 objects, determined by the single most frequently chosen category for each item. Dashes (–) in the MCA column indicate categories that were utilized by participants but never emerged as the most frequent (modal) consensus for any individual object.

Categories	MCA % (N. Objects)	All entries (Entries)
Food	62.12% (82)	58.49% (4015)
Kitchen Utensils	30.30% (40)	29.53% (2027)
Cutlery and Plates	6.06% (8)	5.19% (356)
Unrecognized	0.02% (2)	3.50% (240)
Unknown	–	1.37% (94)
Tools	–	1.12% (77)
Decoration	–	0.67% (46)
Office Supplies	–	0.07% (5)
Cosmetics	–	0.03% (2)
Clothing	–	0.03% (2)

### Descriptive statistics and distributional properties

Descriptive statistics for the cognitive and perceptual dimensions are summarized in Table 6. The analysis revealed distinct distributional profiles across the dimensions.

The dimensions of *Recognition, Name Certainty, Familiarity, Category Certainty,* and *Visual Complexity* exhibited pronounced ceiling effects, with high means (M>91) and medians at the scale maximum of 100. These variables also showed substantial negative skewness (Skewness range: −2.73 to −6.66) and high kurtosis, indicating that the stimulus set comprised predominantly objects that were highly recognizable, familiar, and easily categorized. In contrast, the functional dimensions *Contact* and *Usage* (analyzed prior to merging) displayed greater variability (SD>32) and approximately symmetric distributions (Skewness ≈0). This pattern suggests that, while the objects were generally well-known, the dataset captured a broad spectrum of functional interactions, from objects rarely encountered to those used on a daily basis. To reduce multicollinearity between Contact and Usage (VIFs >9, ρ=.96, p<.001), these variables were averaged to form the composite variable **Object Interaction**, which retained the variability of the original functional dimensions while ensuring statistical independence.

**Table 6:** Descriptive statistics for cognitive and perceptual dimensions.

Dimension	Mean	SD	Median	Skew	Kurtosis
Recognition	96.76	14.10	100.00	−5.31	30.58
Name Certainty	91.21	22.71	100.00	−2.89	7.83
Familiarity	96.71	13.67	100.00	−5.62	34.46
Category Certainty	99.11	4.85	100.00	−6.66	46.84
Visual Complexity	94.56	11.84	100.00	−2.73	7.33
Contact${}^{†}$	62.17	32.45	66.25	−0.42	−1.03
Usage${}^{†}$	46.78	33.92	46.50	0.22	−1.29
**Object Interaction**${}^{‡}$	54.48	32.50	56.38	−0.10	−1.20

*Note.*${}^{†}$Statistics calculated prior to merging to illustrate distribution. ${}^{‡}$Composite variable created by averaging Contact and Usage.

### Correlations between dimensions

**Fig. 6:**
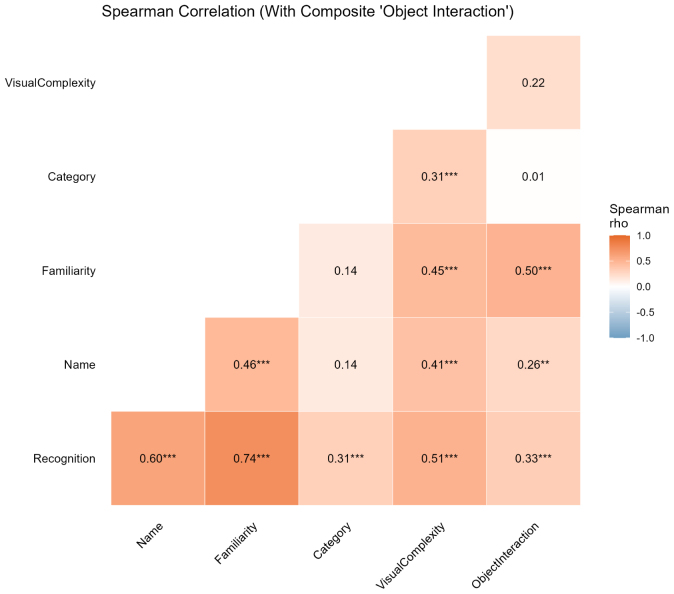
*Spearman rank-order correlation matrix of the final six cognitive and perceptual dimensions.* Color intensity represents the strength and direction of the correlation coefficient (ρ). Asterisks denote statistical significance after Bonferroni correction (* adjusted p<.05; ** adjusted p<.01; *** adjusted p<.001). Non-significant correlations are left blank.

Interrelationships among the six final dimensions are depicted in Fig. 6. Full statistical output, including 95% confidence intervals and Bonferroni-adjusted p-values, is reported in Supplementary Material 4.

*Recognition* emerged as the most interconnected variable. It showed strong positive correlations with **Familiarity** (ρ=.74, p<.001) and **Name Certainty** (ρ=.60, p<.001), indicating that objects that are easily recognized are also rated as more familiar and named with higher confidence.

The composite variable **Object Interaction** exhibited moderate positive correlations with **Familiarity** (ρ=.50, p<.001) and **Recognition** (ρ=.33, p=.002), suggesting that objects frequently used or contacted are also those best recognized and most familiar.

**Category Certainty** displayed a distinct pattern. It was moderately correlated with **Recognition** (ρ=.31, p=.005) and **Visual Complexity** (ρ=.31, p=.005), but unrelated to **Object Interaction** (ρ=.01, p>.999) and **Name Certainty** (ρ=.14, p>.999). This dissociation indicates that the ability to categorize an object, or its perceived visual complexity, does not necessarily predict functional interactions.

### Comparative analysis of object databases

#### Normative sample size comparison

The E-DLL database collected ratings from a normative sample of N=52 participants, which is smaller than those used in several other databases. For example, BDIA 3D (Monteiro et al., 2011) collected ratings from N=214 participants, Peeters (2018) from N=168, and OpenVirtualObjects (Tromp et al., 2020) from N=56. Despite the smaller sample size, E-DLL provides comprehensive coverage across recognition, naming, familiarity, contact, usage, visual complexity, and emotional dimensions, whereas many other databases report only a subset of these measures.

#### Emotional ratings

Across the 132 objects in the E-DLL database, the mean Valence rating was 5.59 ± 0.27, the mean Arousal rating was 2.12 ± 0.17, and the mean Dominance rating was 7.64 ± 0.12. For comparison, the BDIA 3D database (Monteiro et al., 2011), which provides ratings for 131 objects, reported a mean Valence of 4.48 ± 1.73 and a mean Arousal of 3.93 ± 1.28; Dominance was not assessed. E-DLL also includes categorical emotional labels based on Ekman’s Basic Emotions, whereas BDIA 3D does not offer categorical annotations.

#### Stimulus presentation differences

The databases also differ in their stimulus presentation modalities. In the E-DLL, objects were presented as rotating 3D videos, each rotating around its vertical axis at 60°/s for 8 s to ensure full visibility on a 1080p monitor, following the procedure of Tromp et al. (2020). Similarly, Popic et al. (2020) presented virtual objects online one at a time, providing an automatic four-second grayscale video showing all sides of each object, with the option for participants to replay the video. In contrast, the BDIA 3D database (Monteiro et al., 2011) validated its stimuli using static images displayed via a projector-based setup. Meanwhile, Peeters (2018) employed an immersive CAVE projection environment (Cruz-Neira et al., 1993, Gonçalves et al., 2021), which allowed objects to be displayed at real-life size. Together, these methodological differences highlight the substantial heterogeneity in how existing databases convey 3D object information.

#### Dimensional coverage across databases

Table 7, Table 8 summarize the coverage of cognitive, perceptual, and emotional dimensions across datasets. E-DLL is the only database that includes complete ratings for recognition, naming, familiarity, contact, usage, visual complexity, and emotional dimensions (Valence, Arousal, Dominance, and categorical emotion labels). Other databases typically include only a subset of these measures.

#### Object attribute ratings

After mapping all original 1–5 scales to a common 1–100 scale, E-DLL objects demonstrated high recognition (91.83 ± 12.89), high familiarity (88.93 ± 13.35), and high visual complexity (82.32 ± 9.12). Contact (58.94 ± 21.73) and usage (48.64 ± 22.53) ratings fell within the mid-range, and name agreement reached 83.62%. These values are broadly comparable to those reported in OpenVirtualObjects (OVO) (Tromp et al., 2020), as well as in Popic et al. (2020) and Peeters (2018). Notably, E-DLL shows higher visual complexity ratings than those reported in the other databases, whereas its recognition and familiarity scores are most similar to those observed in OVO (Tromp et al., 2020).

**Table 7:** Descriptive Summary of 3D Object Databases.

Database	Objects	N	Recogn	Name	Familiarity	Contact	Usage	Visual Complexity	E-Rating
Monteiro et al. (2011)	131	214							✓
Peeters (2018)	147	168		✓	✓			✓	
Tromp et al. (2020)	124	56	✓	✓	✓	✓	✓	✓	
Popic et al. (2020)	121	83		✓	✓			✓	
**E-DLL (Ours)**	**132**	**52**	✓	✓	✓	✓	✓	✓	✓

*Note.* Presence of a dimension is indicated by a checkmark (✓). Abbreviations: Recogn = Recognition, E-Rating = Emotional Rating. The bold row highlights the most complete database. For Emotional Rating, E-DLL evaluates both dimensional (Valence, Arousal, Dominance, etc.) and categorical frameworks fully, whereas BDIA3D (Monteiro et al., 2011) only evaluated Valence and Arousal.

**Table 8:** Comparison of object attribute ratings across databases (E-DLL, OVO, Popic, and Peeters) after mapping the original 1–5 scale to a 1–100 scale. The table shows the mean ratings with standard deviations for Recognition, Familiarity, Visual Complexity, Contact, and Usage. Name Agreement (NA) is presented as a percentage. N.R. - Not Reported.

Database	Recognition	Familiarity	Visual Complexity	Contact	Usage	NA (%)
**E-DLL (Ours)**	**91.83**±**12.89**	**88.93**±**13.35**	**82.32**±**9.12**	**58.94**±**21.73**	**48.64**±**22.53**	**83.62**±**18.68**
Tromp et al. (2020)	84.63±15.38	81.09±13.79	61.57±16.69	57.15±23.93	50.34±24.64	54.88± N.R.
Popic et al. (2020)	–	84.52±10.37	36.04±11.19	–	–	74.00±22.80
Peeters (2018)	–	55.44±18.57	42.94±15.06	–	–	74.99±22.98

#### Effect size comparisons across databases

To quantify the magnitude of differences between E-DLL and other 3D object databases, Hedges’ g was computed for all overlapping attributes where standard deviations were available. Across comparisons, E-DLL consistently showed higher Visual Complexity, moderate increases in Familiarity and Recognition, and largely similar Contact and Usage ratings relative to existing datasets.

Compared with OpenVirtualObjects (OVO) (Tromp et al., 2020), E-DLL exhibited higher Recognition (g = 0.50) and Familiarity (g = 0.57), and higher Visual Complexity (g = 1.52). Differences in Contact (g = 0.08) and Usage (g =−0.07) were negligible.

Relative to Popic et al. (2020), E-DLL showed slightly higher Familiarity (g = 0.38), substantially higher Visual Complexity (g = 4.41), and moderately higher Name Agreement (g = 0.45).

Comparisons with (Peeters, 2018) revealed higher Familiarity (g = 1.91) and Visual Complexity (g = 2.82).

For emotional ratings, comparisons with BDIA 3D (Monteiro et al., 2011) indicated higher Valence (g = 0.71) and lower Arousal (g =−1.57).

All reported Hedges’ g values indicate standardized differences between E-DLL and the comparison databases. Positive values indicate higher ratings in E-DLL, while negative values indicate lower ratings relative to the comparison dataset.

*Note on Effect Size Interpretation:* Readers should interpret the extremely large effect sizes (e.g., g=4.41) with caution. These magnitudes are highly unusual in behavioral research and primarily reflect a statistical artifact of scale transformations combined with restricted variance in the comparison datasets, rather than massive substantive differences (for a detailed methodological explanation, see Discussion).

## Discussion

The present study provides a comprehensive characterization of the Emotional Daily Life Library (E-DLL), demonstrating its utility for cognitive, perceptual, and clinical research. By integrating emotional, cognitive, and functional dimensions, E-DLL offers a unique resource for investigating how everyday objects are perceived and emotionally interpreted across individuals. Importantly, the results highlight both normative patterns and individual differences, which have implications for experimental design and clinical applications.

### Internal consistency and validity of psychological measures

Our analyses revealed generally good reliability for the NEO-FFI personality factors, closely mirroring the values reported in the European Portuguese validation (Magalhães et al., 2014) with Neuroticism and Conscientiousness demonstrating the highest internal consistency. The Beck Depression Inventory (BDI) demonstrated moderate reliability (α=0.67), which is lower than the excellent theoretical reliability typically observed for the validated Portuguese version (α=0.90–0.91) (Campos & Gonçalves, 2011). This reduction is an expected statistical artifact of restricted variance resulting from the exclusion of participants with higher depressive scores (< 13). The close agreement between Cronbach’s α and McDonald’s ω suggests that these measures reliably captured trait-level variability, supporting their use for modeling individual differences in affective responses to objects (Cronbach, 1951, McDonald, 1999).

### Emotional ratings and individual differences

E-DLL objects elicited predominantly neutral valence, low arousal, and high dominance ratings, reflecting their everyday, non-provocative nature. Neutral stimuli provide substantial experimental advantages, serving as reliable baseline controls in cognitive, perceptual, and clinical paradigms, where unintended emotional activation can confound outcomes (Dolcos et al., 2011, Jefferies et al., 2008, Pereira et al., 2023, Podsakoff et al., 2003, Schweizer et al., 2019).

**Dimensional Ratings (Valence, Arousal, Dominance).** Cumulative Link Mixed Models (CLMMs) revealed that individual differences in personality and depressive symptoms influenced emotional responses (LeMoult & Gotlib, 2019). For valence, higher Neuroticism predicted lower emotional ratings (OR =0.482, p=.044), and this effect was amplified in participants with relatively higher subclinical depressive symptoms, as indicated by the improved fit of the interaction model ($\Delta \chi^{2}\left(1\right)=5.62$, p=.018) and a significant Neuroticism × BDI interaction (OR =0.438, p=.015). For arousal, adding the interaction term did not improve model fit ($\Delta \chi^{2}\left(1\right)=0.01$, p=.906). Instead, strong marginal trends in the main-effects model indicated that higher Neuroticism (OR =3.153, p=.070) and higher Conscientiousness (OR =3.824, p=.053) were associated with higher emotional arousal ratings. Finally, dominance ratings, evaluated via a simplified random-intercept model due to convergence constraints, were not significantly predicted by personality traits or depressive symptoms (interaction OR =0.407, p=.286). However, these null effects should be interpreted with caution, as the dominance ratings exhibited a pronounced ceiling effect (median = 9) and restricted variability, which inherently constrained the statistical sensitivity to detect individual differences.

**Categorical Emotion Attribution.** Analysis of categorical ratings via GLMMs (6864 observations across 52 participants) further elucidated these trait-level influences. While fixed personality traits explained a modest proportion of variance (marginal $R^{2}=0.016$–0.150), specific trait associations emerged for less frequent emotions. The likelihood of attributing Sadness was significantly predicted by both Neuroticism (OR =2.63, p=.048) and Extraversion (OR =2.36, p=.049). The finding regarding Extraversion is particularly noteworthy; while strongly associated with positive affectivity and social potency, higher Extraversion is also linked to a generally heightened sensitivity and faster reactivity to emotional cues in the environment (Rusting, 1998). In the context of neutral, everyday stimuli, which are inherently ambiguous, individuals higher in Extraversion may exhibit a ‘surgency’ in reporting emotional content, leading to a higher likelihood of projecting discrete emotional categories, including Sadness, onto otherwise mundane 3D objects. Furthermore, Fear demonstrated a trending positive association with Neuroticism (OR =2.24, p=.070), and Surprise exhibited a trending negative association with Agreeableness (OR =0.48, p=.052), indicating more agreeable participants were less likely to select this emotion. No significant fixed effects were found for Neutral, Happiness, Anger, or Disgust. The inclusion of random structures substantially increased the total variance explained (conditional $R^{2}=0.537$–0.742), highlighting that emotion attribution shifts dynamically between subjective response tendencies (for high-frequency categories like Neutral and Happiness) and specific stimulus properties. However, it is important to note that the extremely low base rates of negative emotions result in sparse data, which naturally limits the stability of variance partitioning at the stimulus level for these specific categories.

Together, these findings show that individual differences impact emotional perception in two different ways. For dimensional ratings (Valence, Arousal, Dominance), the ratings are highly sensitive to the person’s personality and mood, especially the combination of Neuroticism and depressive symptoms. For categorical labels, however, the results are mostly determined by the objects themselves; for example, most people agree on which objects are “Neutral” regardless of their personality. The exception occurs with negative emotions like Sadness or Fear: even though these reactions are rare, they are significantly predicted by traits like Neuroticism and Extraversion. Ultimately, this underscores the importance of accounting for both the properties of the objects and the personality of the participants when using everyday 3D stimuli in experimental or clinical settings.

### Comparison with existing 3D object databases

E-DLL distinguishes itself from existing 3D object databases in several key ways. While databases such as BDIA 3D (Monteiro et al., 2011), Peeters (2018), and OpenVirtualObjects (Tromp et al., 2020) often include larger normative samples, they frequently provide only partial coverage of cognitive, functional, or emotional dimensions. For instance, BDIA 3D emphasizes Valence and Arousal ratings but lacks comprehensive perceptual and functional annotations. E-DLL, in contrast, captures recognition, naming, familiarity, visual complexity, object interaction, and both dimensional and categorical emotional ratings, offering an integrated resource for multi-faceted research.

Effect size analyses (Hedges’ g) further quantify the magnitude of differences between E-DLL and these other databases (Note: Positive g values indicate higher ratings in E-DLL, whereas negative g values indicate lower ratings relative to the comparison dataset). Compared with OVO, E-DLL shows moderate increases in Recognition (g = 0.50) and Familiarity (g = 0.57), and a large increase in Visual Complexity (g = 1.52), while differences in Contact and Usage are negligible. Relative to Popic et al. (2020), E-DLL exhibits higher Familiarity (g = 0.38), dramatically greater Visual Complexity (g = 4.41), and moderately higher Name Agreement (g = 0.45). However, readers should interpret these extremely large effect sizes with caution. As detailed in our methodology regarding scale transformations, these magnitudes are primarily a statistical artifact of scale transformations combined with restricted variance. Because the original comparison datasets utilized 1–5 scales with very small standard deviations, mathematically transforming them to a 1–100 metric preserved this tight clustering. Consequently, even modest absolute differences in means between the databases resulted in artificially inflated standardized effect sizes (Borenstein et al., 2021). These extreme values should therefore be understood as reflecting scaling artifacts and distributional differences rather than massive substantive shifts in visual perception. Comparisons with Peeters (2018) show large increases in both Familiarity (g = 1.91) and Visual Complexity (g = 2.82). Emotional comparisons with BDIA 3D indicate medium increases in Valence (g = 0.71) and large decreases in Arousal (g =−1.57), reflecting the neutral, low-arousal nature of E-DLL stimuli. Collectively, these standardized effect sizes underscore that E-DLL not only provides broader dimensional coverage but also introduces meaningful differences in perceptual and emotional characteristics, particularly in Visual Complexity, Name Agreement, and emotional neutrality.

Moreover, the predominance of neutral, low-arousal stimuli sets E-DLL apart from databases that focus on eliciting strong affective responses. Neutral objects are particularly valuable for clinical research, where controlling for emotional confounds is critical in cognitive assessments, affective bias tasks, and intervention studies (Dolcos et al., 2011, Podsakoff et al., 2003). The standardized rotating 3D video presentation further enhances ecological validity by providing full object visibility, a methodological advantage over static images or partial-object presentations.

Overall, the combined coverage of perceptual, functional, and emotional dimensions, along with the systematic increase in Visual Complexity and moderate improvements in Familiarity and Name Agreement, highlight the utility of E-DLL as a versatile and controlled stimulus set for cognitive, affective, and clinical research.

### Clinical implications

The present findings highlight the utility of E-DLL for prospective clinical applications, particularly in mental health research and cognitive rehabilitation. Neutral, low-arousal objects are highly valuable in clinical paradigms, as they facilitate repeated-measures designs and minimize affective contamination while maintaining strong ecological validity. Furthermore, the 3D rotating format aligns experimental tasks more closely with real-life object interaction than static 2D images. While widespread clinical application of this database remains prospective, preliminary implementations already demonstrate its practical potential. For example, E-DLL stimuli have been successfully integrated into platforms such as NeuroAIreh@b, which leverages artificial intelligence to personalize neurorehabilitation tasks based on simulated daily life activities (Câmara et al., 2025, Câmara et al., 2024, Faria et al., 2024, Paulino et al., 2022). Crucially, our validation data regarding individual differences has direct implications for how these stimuli should be interpreted in clinical settings. The observed affective bias—whereby individuals with higher Neuroticism and subclinical depressive symptoms attributed lower valence and discrete negative emotions to neutral objects—emphasizes the need to account for trait-level affective tendencies. Practitioners and researchers should consider that variations in emotional response or task engagement with these everyday stimuli may reflect underlying mood states rather than primary cognitive processing differences. By providing a multidimensional, validated baseline, E-DLL allows researchers to systematically control for these affective biases, bridging the gap between experimental rigor and prospective clinical application.

### Limitations and future directions

Several limitations should be noted. First, while the total observation count (N=6864) provided robust power for estimating variance components and stimulus-level effects, the participant-level sample size (N=52) is relatively modest. To transparently assess the impact of this constraint within our mixed-modeling framework, we conducted a 10,000-iteration Monte Carlo simulation-based sensitivity analysis (via cluster bootstrapping). This empirical test revealed an estimated probability of 64.09% to detect the critical Neuroticism × BDI interaction for Valence, and 58.46% to detect the main effect of Neuroticism. Therefore, while these simulations confirm that the model estimations are statistically stable, the participant-level sample size limits our design’s overall sensitivity. Consequently, the magnitude of the reported odds ratios should be interpreted with appropriate caution, and our ability to detect more subtle between-subject individual differences may be constrained.

Second, while the Emotional Daily Life Library provides a robust normative baseline, the sampling criteria used in this validation introduce certain boundaries. To ensure that baseline emotional ratings were not systematically skewed by clinical affective biases, we explicitly excluded participants with BDI-II scores indicative of mild-to-moderate depression (> 13). Consequently, the variance of depressive symptomatology within our sample was inherently restricted, which limits our ability to fully model affective bias across the clinical spectrum. However, retaining this subclinical variance allowed us to demonstrate that even minor, non-clinical fluctuations in mood interact meaningfully with trait Neuroticism to modulate emotional appraisal. Therefore, while these findings highlight the sensitivity of our 3D objects to individual differences, the observed trait-mood interactions should be interpreted strictly within the context of a healthy population. Future studies utilizing this database should investigate affective appraisals across full clinical samples to comprehensively model how moderate and severe depressive symptoms bias the perception of everyday emotional objects.

Third, while the database was explicitly designed with virtual reality (VR) and immersive applications in mind, it is important to clarify the boundaries of the current study’s ecological validity. Because the present validation was conducted using rotating 2D videos on standard monitors rather than fully immersive headsets, the ecological relevance of this database currently derives from the highly realistic, everyday nature of the 3D objects themselves, rather than an immersive testing environment. However, presenting the objects in continuous rotation allowed participants to perceive structural depth and interaction affordances more naturally than traditional static images, successfully bridging the gap between 2D databases and fully interactive environments. Future research should build upon this foundational normative validation by evaluating these stimuli directly within immersive VR settings to determine how fully interactive spatial presence might further modulate emotional appraisal.

Fourth, a methodological caveat must be noted regarding the sparse base rates observed for specific negative categorical emotions (e.g., Anger, Sadness). Because our categorical outcomes were binary, the extremely low frequency of negative emotional attributions toward neutral everyday objects naturally constrained our ability to reliably partition variance at the stimulus level. In mixed-effects modeling, such sparse data can lead to unstable variance components when fitting maximal random-effects structures. Consequently, while we successfully modeled the fixed effects of personality traits using GLMMs with simplified random-effects structures, our ability to interpret object-level variance for these rare negative emotions is limited. Future studies aiming to isolate stimulus-driven variance for low-frequency emotions should consider utilizing larger, highly valenced stimulus sets or alternative forced-choice paradigms.

Finally, some objects exhibited lower recognition or naming agreement, suggesting that cultural or experiential familiarity may influence perception. Future work should expand the normative sample and include cross-cultural validation to enhance the global applicability of the E-DLL stimuli.

### Conclusions

E-DLL provides a highly controlled, multidimensional set of everyday objects with robust perceptual, functional, and emotional characterization. Neutral stimuli dominate the database, enabling baseline comparisons and reducing affective confounds in clinical and experimental paradigms. Individual differences in personality and depressive symptoms shape subtle interpretations of these objects, providing insights into affective biases relevant for clinical populations.

Compared with existing 3D object databases, E-DLL not only offers the most comprehensive coverage of cognitive, functional, and emotional dimensions, but also differs meaningfully in key attributes. Effect size analyses indicate moderate to large increases in Visual Complexity and Familiarity relative to OpenVirtualObjects, Popic, and Peeters, as well as medium increases in Valence and large decreases in Arousal relative to BDIA 3D. These standardized differences highlight that E-DLL stimuli are perceptually richer and emotionally more neutral than objects in other databases.

Overall, E-DLL constitutes a versatile, methodologically rigorous resource for affective science, cognitive neuroscience, and clinical research, supporting controlled, ecologically valid experimental and clinical applications.

## CRediT authorship contribution statement

**Diogo Branco:** Conceptualization, Methodology, Programming, Data curation, Data analysis, Writing – original draft, Writing – review & editing. **Mariana Castro Fernandes:** Data curation, Review. **Sergi Bermúdez i Badia:** Methodology, Project administration, Supervision, Review & editing. **Ana Lúcia Faria:** Methodology, Project administration, Supervision, Review & editing.

## Funding

This work was supported by Fundação para a Ciência e Tecnologia (FCT) under the PhD grant of Diogo Branco with reference 2021.05646.BD ( https://doi.org/10.54499/2021.05646.BD) and by NOVA Laboratory for Computer Science and Informatics (UID/04516/2025) with the financial support of FCT.IP ( https://doi.org/10.54499/UID/04516/2025).

## Declaration of competing interest

The authors declare that they have no known competing financial interests or personal relationships that could have appeared to influence the work reported in this paper.

## Data Availability

All data and objects, along with their corresponding ratings, screenshots, and videos used in this validation, are available online at the Open Source Framework repository at https://osf.io/98hk2/. Additionally, license and credits for each object are provided. Please contact the corresponding author for further inquiries.

## Supplementary data

MMC S1 Detailed statistical models for emotions and affect, alongside correlations for cognitive-perceptual dimensions. MMC S1

MMC S2

MMC S3

MMC S4
