# Supplementary material for: Emotional Daily Life Library (E-DLL): Validation of a database of 3D objects for emotion elicitation
Source: Int J Clin Health Psychol. 2026 May 14;26(2):100690. doi: 10.1016/j.ijchp.2026.100690 (PMC13202292; doi:10.1016/j.ijchp.2026.100690)
Supplement: MMC S1 — Detailed statistical models for emotions and affect, alongside correlations for cognitive-perceptual dimensions. [file mmc1.pdf]

Supplementary Material 1: CLMM Results (Object_ID as Crossed Effect)

Model	Predictor	OR	CI_Low	CI_High	p_value	Max_Grad	AIC	BIC
Valence (Main)	Z_Neuro	0.482	0.237	0.980	0.0438	0.00028	9,992.4	10,074.4
Valence (Main)	Z_Extra	0.775	0.385	1.561	0.4761	0.00028	9,992.4	10,074.4
Valence (Main)	Z_Open	1.053	0.543	2.043	0.8788	0.00028	9,992.4	10,074.4
Valence (Main)	Z_Agree	0.833	0.393	1.768	0.6347	0.00028	9,992.4	10,074.4
Valence (Main)	Z_Conc	0.565	0.267	1.198	0.1367	0.00028	9,992.4	10,074.4
Valence (Main)	Z_BDI	0.866	0.455	1.646	0.6598	0.00028	9,992.4	10,074.4
Valence (Int)	Z_Neuro	0.576	0.289	1.146	0.1162	0.00571	9,988.8	10,077.7
Valence (Int)	Z_BDI	0.692	0.366	1.307	0.2562	0.00571	9,988.8	10,077.7
Valence (Int)	Z_Extra	0.766	0.394	1.488	0.4309	0.00571	9,988.8	10,077.7
Valence (Int)	Z_Open	0.885	0.465	1.687	0.7107	0.00571	9,988.8	10,077.7
Valence (Int)	Z_Agree	0.766	0.374	1.569	0.4659	0.00571	9,988.8	10,077.7
Valence (Int)	Z_Conc	0.755	0.357	1.598	0.4624	0.00571	9,988.8	10,077.7
Valence (Int)	Z_Neuro:Z_BDI	0.438	0.225	0.853	0.0151	0.00571	9,988.8	10,077.7
Arousal (Main)	Z_Neuro	3.153	0.909	10.935	0.0703	0.00082	6,241.5	6,323.5
Arousal (Main)	Z_Extra	0.658	0.200	2.159	0.4895	0.00082	6,241.5	6,323.5
Arousal (Main)	Z_Open	0.736	0.237	2.285	0.5963	0.00082	6,241.5	6,323.5
Arousal (Main)	Z_Agree	0.457	0.116	1.797	0.2623	0.00082	6,241.5	6,323.5
Arousal (Main)	Z_Conc	3.824	0.982	14.886	0.0531	0.00082	6,241.5	6,323.5
Arousal (Main)	Z_BDI	1.441	0.467	4.448	0.5251	0.00082	6,241.5	6,323.5
Arousal (Int)	Z_Neuro	3.099	0.866	11.093	0.0821	0.00085	6,243.5	6,332.3
Arousal (Int)	Z_BDI	1.464	0.460	4.656	0.5183	0.00085	6,243.5	6,332.3
Arousal (Int)	Z_Extra	0.657	0.200	2.155	0.4879	0.00085	6,243.5	6,332.3
Arousal (Int)	Z_Open	0.749	0.233	2.404	0.6271	0.00085	6,243.5	6,332.3
Arousal (Int)	Z_Agree	0.461	0.117	1.825	0.2703	0.00085	6,243.5	6,332.3
Arousal (Int)	Z_Conc	3.713	0.876	15.727	0.0749	0.00085	6,243.5	6,332.3
Arousal (Int)	Z_Neuro:Z_BDI	1.077	0.316	3.671	0.9059	0.00085	6,243.5	6,332.3
Dominance (Main)	Z_Neuro	0.330	0.059	1.860	0.209	0.00023	5,260.5	5,335.7
Dominance (Main)	Z_Extra	1.408	0.271	7.324	0.684	0.00023	5,260.5	5,335.7
Dominance (Main)	Z_Open	1.138	0.233	5.568	0.873	0.00023	5,260.5	5,335.7
Dominance (Main)	Z_Agree	1.954	0.303	12.587	0.481	0.00023	5,260.5	5,335.7
Dominance (Main)	Z_Conc	0.708	0.117	4.281	0.707	0.00023	5,260.5	5,335.7
Dominance (Main)	Z_BDI	1.469	0.299	7.212	0.635	0.00023	5,260.5	5,335.7
Dominance (Int)	Z_Neuro	0.397	0.072	2.180	0.287	0.00008	5,261.4	5,343.4
Dominance (Int)	Z_BDI	1.214	0.247	5.963	0.811	0.00008	5,261.4	5,343.4
Dominance (Int)	Z_Extra	1.441	0.288	7.223	0.657	0.00008	5,261.4	5,343.4
Dominance (Int)	Z_Open	0.948	0.191	4.702	0.947	0.00008	5,261.4	5,343.4
Dominance (Int)	Z_Agree	1.744	0.279	10.905	0.552	0.00008	5,261.4	5,343.4
Dominance (Int)	Z_Conc	1.008	0.154	6.595	0.993	0.00008	5,261.4	5,343.4
Dominance (Int)	Z_Neuro:Z_BDI	0.407	0.078	2.122	0.286	0.00008	5,261.4	5,343.4
