# Supplementary material for: Emotional Daily Life Library (E-DLL): Validation of a database of 3D objects for emotion elicitation
Source: Int J Clin Health Psychol. 2026 May 14;26(2):100690. doi: 10.1016/j.ijchp.2026.100690 (PMC13202292; doi:10.1016/j.ijchp.2026.100690)
Supplement: MMC S2 [file mmc2.pdf]

Supplementary Material 2: Generalized Linear Mixed Models (GLMM)

Supplementary Table S2.1. Combined summary of emotion models. Odds ratios (OR) with 95% confidence intervals (CI), significance stars, and model fit statistics (AIC, BIC, Marginal and Conditional R^2) are reported for all predictors across all emotions. Note: Predictors were scaled prior to analysis; thus, ORs represent a 1-SD increase in the predictor. To ensure model parsimony and stability, the video-level random effect was excluded from models where likelihood ratio tests indicated it did not significantly improve model fit (e.g., Anger, Sadness).

Emotion	term	OR_CI_sig	p-value	AIC	BIC	Marginal_R2	Conditional_R2
Neutral	(Intercept)	26.193 (12.109-56.66)***	< 0.001	4,026.837	4,088.344	0.048	0.697
Neutral	BDI_Score	0.723 (0.336-1.552)	0.405	4,026.837	4,088.344	0.048	0.697
Neutral	Neuroticism_Factor	0.617 (0.27-1.414)	0.254	4,026.837	4,088.344	0.048	0.697
Neutral	Extraversion_Factor	1.031 (0.463-2.295)	0.940	4,026.837	4,088.344	0.048	0.697
Neutral	OpennessToExperience_Factor	0.742 (0.343-1.605)	0.449	4,026.837	4,088.344	0.048	0.697
Neutral	Agreeableness_Factor	1.423 (0.572-3.54)	0.448	4,026.837	4,088.344	0.048	0.697
Neutral	Conscientiousness_Factor	0.903 (0.371-2.195)	0.822	4,026.837	4,088.344	0.048	0.697
Happiness	(Intercept)	0.021 (0.01-0.042)***	< 0.001	3,248.141	3,309.647	0.016	0.661
Happiness	BDI_Score	1.09 (0.564-2.106)	0.797	3,248.141	3,309.647	0.016	0.661
Happiness	Neuroticism_Factor	1.176 (0.573-2.413)	0.659	3,248.141	3,309.647	0.016	0.661
Happiness	Extraversion_Factor	0.751 (0.376-1.499)	0.417	3,248.141	3,309.647	0.016	0.661
Happiness	OpennessToExperience_Factor	1.192 (0.615-2.31)	0.602	3,248.141	3,309.647	0.016	0.661
Happiness	Agreeableness_Factor	0.994 (0.45-2.192)	0.987	3,248.141	3,309.647	0.016	0.661
Happiness	Conscientiousness_Factor	1.158 (0.532-2.524)	0.711	3,248.141	3,309.647	0.016	0.661
Fear	(Intercept)	0 (0-0.002)***	< 0.001	618.664	680.170	0.096	0.692
Fear	BDI_Score	1.357 (0.634-2.906)	0.432	618.664	680.170	0.096	0.692
Fear	Neuroticism_Factor	2.235 (0.936-5.339)	0.070	618.664	680.170	0.096	0.692
Fear	Extraversion_Factor	1.038 (0.489-2.204)	0.923	618.664	680.170	0.096	0.692
Fear	OpennessToExperience_Factor	1.697 (0.783-3.677)	0.180	618.664	680.170	0.096	0.692
Fear	Agreeableness_Factor	0.845 (0.331-2.157)	0.725	618.664	680.170	0.096	0.692
Fear	Conscientiousness_Factor	1.045 (0.435-2.515)	0.921	618.664	680.170	0.096	0.692
Surprise	(Intercept)	0.015 (0.008-0.03)***	< 0.001	2,670.156	2,731.663	0.150	0.626
Surprise	BDI_Score	0.997 (0.533-1.865)	0.991	2,670.156	2,731.663	0.150	0.626
Surprise	Neuroticism_Factor	1.635 (0.834-3.204)	0.152	2,670.156	2,731.663	0.150	0.626
Surprise	Extraversion_Factor	0.961 (0.506-1.825)	0.904	2,670.156	2,731.663	0.150	0.626
Surprise	OpennessToExperience_Factor	1.207 (0.649-2.246)	0.552	2,670.156	2,731.663	0.150	0.626
Surprise	Agreeableness_Factor	0.476 (0.225-1.008)	0.052	2,670.156	2,731.663	0.150	0.626
Surprise	Conscientiousness_Factor	0.743 (0.365-1.51)	0.411	2,670.156	2,731.663	0.150	0.626
Anger	(Intercept)	0.001 (0-0.004)***	< 0.001	639.652	694.324	0.116	0.742
Anger	BDI_Score	1.276 (0.457-3.558)	0.642	639.652	694.324	0.116	0.742
Anger	Neuroticism_Factor	1.027 (0.331-3.181)	0.963	639.652	694.324	0.116	0.742
Anger	Extraversion_Factor	0.955 (0.33-2.764)	0.932	639.652	694.324	0.116	0.742
Anger	OpennessToExperience_Factor	1.524 (0.534-4.351)	0.431	639.652	694.324	0.116	0.742
Anger	Agreeableness_Factor	0.367 (0.094-1.435)	0.150	639.652	694.324	0.116	0.742
Anger	Conscientiousness_Factor	0.657 (0.203-2.12)	0.482	639.652	694.324	0.116	0.742
Sadness	(Intercept)	0.002 (0.001-0.005)***	< 0.001	692.162	746.834	0.139	0.598
Sadness	BDI_Score	1.199 (0.556-2.585)	0.643	692.162	746.834	0.139	0.598
Sadness	Neuroticism_Factor	2.63 (1.007-6.865)*	0.048	692.162	746.834	0.139	0.598
Sadness	Extraversion_Factor	2.359 (1.004-5.539)*	0.049	692.162	746.834	0.139	0.598

Emotion	term	OR_CI_sig	p-value	AIC	BIC	Marginal_R2	Conditional_R2
Sadness	OpennessToExperience_Factor	0.888 (0.403-1.956)	0.769	692.162	746.834	0.139	0.598
Sadness	Agreeableness_Factor	0.713 (0.293-1.734)	0.455	692.162	746.834	0.139	0.598
Sadness	Conscientiousness_Factor	1.408 (0.599-3.314)	0.433	692.162	746.834	0.139	0.598
Disgust	(Intercept)	0.011 (0.007-0.02)***	< 0.001	2,094.432	2,155.938	0.025	0.537
Disgust	BDI_Score	1.109 (0.685-1.793)	0.674	2,094.432	2,155.938	0.025	0.537
Disgust	Neuroticism_Factor	1.421 (0.834-2.42)	0.196	2,094.432	2,155.938	0.025	0.537
Disgust	Extraversion_Factor	1.094 (0.661-1.813)	0.726	2,094.432	2,155.938	0.025	0.537
Disgust	OpennessToExperience_Factor	1.044 (0.641-1.702)	0.861	2,094.432	2,155.938	0.025	0.537
Disgust	Agreeableness_Factor	0.721 (0.403-1.291)	0.271	2,094.432	2,155.938	0.025	0.537
Disgust	Conscientiousness_Factor	1.274 (0.722-2.249)	0.403	2,094.432	2,155.938	0.025	0.537

Supplementary Table S2.2. Detailed GLMM results for the Neutral model.

term	estimate	std.error	statistic	p-value	OR_CI_sig	AIC	BIC	Marginal_R2	Conditional_R2
(Intercept)	3.266	0.394	8.295	< 0.001	26.193 (12.109-56.66)***	4,026.837	4,088.344	0.048	0.697
BDI_Score	-0.325	0.390	-0.833	0.405	0.723 (0.336-1.552)	4,026.837	4,088.344	0.048	0.697
Neuroticism_Factor	-0.482	0.423	-1.140	0.254	0.617 (0.27-1.414)	4,026.837	4,088.344	0.048	0.697
Extraversion_Factor	0.031	0.408	0.075	0.940	1.031 (0.463-2.295)	4,026.837	4,088.344	0.048	0.697
OpennessToExperience_Factor	-0.298	0.393	-0.758	0.449	0.742 (0.343-1.605)	4,026.837	4,088.344	0.048	0.697
Agreeableness_Factor	0.353	0.465	0.759	0.448	1.423 (0.572-3.54)	4,026.837	4,088.344	0.048	0.697
Conscientiousness_Factor	-0.102	0.453	-0.225	0.822	0.903 (0.371-2.195)	4,026.837	4,088.344	0.048	0.697

Supplementary Table S2.3. Detailed GLMM results for the Happiness model.

term	estimate	std.error	statistic	p-value	OR_CI_sig	AIC	BIC	Marginal_R2	Conditional_R2
(Intercept)	-3.867	0.356	-10.871	< 0.001	0.021 (0.01-0.042)***	3,248.141	3,309.647	0.016	0.661
BDI_Score	0.086	0.336	0.257	0.797	1.09 (0.564-2.106)	3,248.141	3,309.647	0.016	0.661
Neuroticism_Factor	0.162	0.367	0.441	0.659	1.176 (0.573-2.413)	3,248.141	3,309.647	0.016	0.661
Extraversion_Factor	-0.286	0.353	-0.812	0.417	0.751 (0.376-1.499)	3,248.141	3,309.647	0.016	0.661
OpennessToExperience_Factor	0.176	0.338	0.521	0.602	1.192 (0.615-2.31)	3,248.141	3,309.647	0.016	0.661
Agreeableness_Factor	-0.006	0.404	-0.016	0.987	0.994 (0.45-2.192)	3,248.141	3,309.647	0.016	0.661
Conscientiousness_Factor	0.147	0.397	0.370	0.711	1.158 (0.532-2.524)	3,248.141	3,309.647	0.016	0.661

Supplementary Table S2.4. Detailed GLMM results for the Fear model.

term	estimate	std.error	statistic	p-value	OR_CI_sig	AIC	BIC	Marginal_R2	Conditional_R2
(Intercept)	-7.651	0.636	-12.033	< 0.001	0 (0-0.002)***	618.664	680.17	0.096	0.692
BDI_Score	0.305	0.389	0.785	0.432	1.357 (0.634-2.906)	618.664	680.17	0.096	0.692
Neuroticism_Factor	0.804	0.444	1.810	0.070	2.235 (0.936-5.339)	618.664	680.17	0.096	0.692
Extraversion_Factor	0.037	0.384	0.097	0.923	1.038 (0.489-2.204)	618.664	680.17	0.096	0.692
OpennessToExperience_Factor	0.529	0.395	1.341	0.180	1.697 (0.783-3.677)	618.664	680.17	0.096	0.692
Agreeableness_Factor	-0.168	0.478	-0.351	0.725	0.845 (0.331-2.157)	618.664	680.17	0.096	0.692
Conscientiousness_Factor	0.044	0.448	0.099	0.921	1.045 (0.435-2.515)	618.664	680.17	0.096	0.692

Supplementary Table S2.5. Detailed GLMM results for the Surprise model.

term	estimate	std.error	statistic	p-value	OR_CI_sig	AIC	BIC	Marginal_R2	Conditional_R2
(Intercept)	-4.182	0.338	-12.363	< 0.001	0.015 (0.008-0.03)***	2,670.156	2,731.663	0.15	0.626
BDI_Score	-0.003	0.320	-0.011	0.991	0.997 (0.533-1.865)	2,670.156	2,731.663	0.15	0.626
Neuroticism_Factor	0.492	0.343	1.433	0.152	1.635 (0.834-3.204)	2,670.156	2,731.663	0.15	0.626
Extraversion_Factor	-0.040	0.327	-0.121	0.904	0.961 (0.506-1.825)	2,670.156	2,731.663	0.15	0.626
OpennessToExperience_Factor	0.188	0.317	0.595	0.552	1.207 (0.649-2.246)	2,670.156	2,731.663	0.15	0.626
Agreeableness_Factor	-0.743	0.383	-1.940	0.052	0.476 (0.225-1.008)	2,670.156	2,731.663	0.15	0.626
Conscientiousness_Factor	-0.298	0.362	-0.822	0.411	0.743 (0.365-1.51)	2,670.156	2,731.663	0.15	0.626

Supplementary Table S2.6. Detailed GLMM results for the Anger model.

term	estimate	std.error	statistic	p-value	OR_CI_sig	AIC	BIC	Marginal_R2	Conditional_R2
(Intercept)	-7.553	1.030	-7.336	< 0.001	0.001 (0-0.004)***	639.652	694.324	0.116	0.742
BDI_Score	0.244	0.523	0.465	0.642	1.276 (0.457-3.558)	639.652	694.324	0.116	0.742
Neuroticism_Factor	0.027	0.577	0.046	0.963	1.027 (0.331-3.181)	639.652	694.324	0.116	0.742
Extraversion_Factor	-0.046	0.542	-0.085	0.932	0.955 (0.33-2.764)	639.652	694.324	0.116	0.742
OpennessToExperience_Factor	0.421	0.535	0.787	0.431	1.524 (0.534-4.351)	639.652	694.324	0.116	0.742
Agreeableness_Factor	-1.003	0.696	-1.441	0.150	0.367 (0.094-1.435)	639.652	694.324	0.116	0.742
Conscientiousness_Factor	-0.421	0.598	-0.703	0.482	0.657 (0.203-2.12)	639.652	694.324	0.116	0.742

Supplementary Table S2.7. Detailed GLMM results for the Sadness model.

term	estimate	std.error	statistic	p-value	OR_CI_sig	AIC	BIC	Marginal_R2	Conditional_R2
(Intercept)	-6.473	0.569	-11.376	< 0.001	0.002 (0.001-0.005)***	692.162	746.834	0.139	0.598
BDI_Score	0.182	0.392	0.464	0.643	1.199 (0.556-2.585)	692.162	746.834	0.139	0.598
Neuroticism_Factor	0.967	0.490	1.975	0.048	2.63 (1.007-6.865)*	692.162	746.834	0.139	0.598
Extraversion_Factor	0.858	0.436	1.970	0.049	2.359 (1.004-5.539)*	692.162	746.834	0.139	0.598
OpennessToExperience_Factor	-0.118	0.403	-0.294	0.769	0.888 (0.403-1.956)	692.162	746.834	0.139	0.598
Agreeableness_Factor	-0.339	0.453	-0.747	0.455	0.713 (0.293-1.734)	692.162	746.834	0.139	0.598
Conscientiousness_Factor	0.342	0.437	0.784	0.433	1.408 (0.599-3.314)	692.162	746.834	0.139	0.598

Supplementary Table S2.8. Detailed GLMM results for the Disgust model.

term	estimate	std.error	statistic	p-value	OR_CI_sig	AIC	BIC	Marginal_R2	Conditional_R2
(Intercept)	-4.470	0.286	-15.643	< 0.001	0.011 (0.007-0.02)***	2,094.432	2,155.938	0.025	0.537
BDI_Score	0.103	0.245	0.420	0.674	1.109 (0.685-1.793)	2,094.432	2,155.938	0.025	0.537
Neuroticism_Factor	0.351	0.272	1.294	0.196	1.421 (0.834-2.42)	2,094.432	2,155.938	0.025	0.537
Extraversion_Factor	0.090	0.258	0.350	0.726	1.094 (0.661-1.813)	2,094.432	2,155.938	0.025	0.537
OpennessToExperience_Factor	0.044	0.249	0.175	0.861	1.044 (0.641-1.702)	2,094.432	2,155.938	0.025	0.537
Agreeableness_Factor	-0.327	0.297	-1.100	0.271	0.721 (0.403-1.291)	2,094.432	2,155.938	0.025	0.537
Conscientiousness_Factor	0.242	0.290	0.836	0.403	1.274 (0.722-2.249)	2,094.432	2,155.938	0.025	0.537

Supplementary Material 2b: Sensitivity Analysis (Likelihood Ratio Tests)

To ensure model parsimony and assess boundary issues, a sensitivity analysis was conducted for each emotion. The full model (crossed random effects for Participant_ID and Object_ID) was compared against a reduced model (Participant_ID random effect only) using Likelihood Ratio Tests (LRTs). Models where the Object_ID random effect did not significantly improve fit ($p > 0.05$) were reduced to prevent statistical separation.

Emotion: Neutral

Singular Fit (Boundary Issue in Full Model): FALSE

Model	Df	AIC	BIC	logLik	deviance	Chisq	Chi Df	Pr(>Chisq)
mod_reduced	8	4,165.84	4,220.51	-2,074.92	4,149.84			
mod_full	9	4,026.84	4,088.34	-2,004.42	4,008.84	141	1	< 0.001

Emotion: Happiness

Singular Fit (Boundary Issue in Full Model): FALSE

Model	Df	AIC	BIC	logLik	deviance	Chisq	Chi Df	Pr(>Chisq)
mod_reduced	8	3,601.76	3,656.43	-1,792.88	3,585.76			
mod_full	9	3,248.14	3,309.65	-1,615.07	3,230.14	355.62	1	< 0.001

Emotion: Fear

Singular Fit (Boundary Issue in Full Model): FALSE

Model	Df	AIC	BIC	logLik	deviance	Chisq	Chi Df	Pr(>Chisq)
mod_reduced	8	681.00	735.67	-332.50	665.00			
mod_full	9	618.66	680.17	-300.33	600.66	64.34	1	< 0.001

Emotion: Surprise

Singular Fit (Boundary Issue in Full Model): FALSE

Model	Df	AIC	BIC	logLik	deviance	Chisq	Chi Df	Pr(>Chisq)
mod_reduced	8	2,693.42	2,748.09	-1,338.71	2,677.42			
mod_full	9	2,670.16	2,731.66	-1,326.08	2,652.16	25.26	1	< 0.001

Emotion: Anger

Singular Fit (Boundary Issue in Full Model): FALSE

Model	Df	AIC	BIC	logLik	deviance	Chisq	Chi Df	Pr(>Chisq)
mod_reduced	8	639.65	694.32	-311.83	623.65			
mod_full	9	639.69	701.20	-310.85	621.69	1.96	1	0.162

Emotion: Sadness

Singular Fit (Boundary Issue in Full Model): FALSE

Model	Df	AIC	BIC	logLik	deviance	Chisq	Chi Df	Pr(>Chisq)
mod_reduced	8	692.16	746.83	-338.08	676.16			
mod_full	9	693.92	755.43	-337.96	675.92	0.24	1	0.624

Emotion: Disgust

Singular Fit (Boundary Issue in Full Model): FALSE

Model	Df	AIC	BIC	logLik	deviance	Chisq	Chi Df	Pr(>Chisq)
mod_reduced	8	2,224.10	2,278.77	-1,104.05	2,208.10			
mod_full	9	2,094.43	2,155.94	-1,038.22	2,076.43	131.67	1	< 0.001
