# Supplementary material for: Emotional Daily Life Library (E-DLL): Validation of a database of 3D objects for emotion elicitation
Source: Int J Clin Health Psychol. 2026 May 14;26(2):100690. doi: 10.1016/j.ijchp.2026.100690 (PMC13202292; doi:10.1016/j.ijchp.2026.100690)
Supplement: MMC S3 [file mmc3.pdf]

Supplementary Material 3: Summary of Emotion Models

	Neutral			Happiness			Fear			Surprise			Anger			Sadness			Disgust		
Predictors	*Odds Ratios*	*CI*	*p*	*Odds Ratios*	*CI*	*p*	*Odds Ratios*	*CI*	*p*	*Odds Ratios*	*CI*	*p*	*Odds Ratios*	*CI*	*p*	*Odds Ratios*	*CI*	*p*	*Odds Ratios*	*CI*	*p*
(Intercept)	26.19	12.11 – 56.66	**<0.001**	0.02	0.01 – 0.04	**<0.001**	0.00	0.00 – 0.00	**<0.001**	0.02	0.01 – 0.03	**<0.001**	0.00	0.00 – 0.00	**<0.001**	0.00	0.00 – 0.00	**<0.001**	0.01	0.01 – 0.02	**<0.001**
BDI Score	0.72	0.34 – 1.55	0.405	1.09	0.56 – 2.11	0.797	1.36	0.63 – 2.91	0.432	1.00	0.53 – 1.86	0.991	1.28	0.46 – 3.56	0.642	1.20	0.56 – 2.58	0.643	1.11	0.69 – 1.79	0.674
Neuroticism Factor	0.62	0.27 – 1.41	0.254	1.18	0.57 – 2.41	0.659	2.24	0.94 – 5.34	0.070	1.64	0.83 – 3.20	0.152	1.03	0.33 – 3.18	0.963	2.63	1.01 – 6.86	**0.048**	1.42	0.83 – 2.42	0.196
Extraversion Factor	1.03	0.46 – 2.29	0.940	0.75	0.38 – 1.50	0.417	1.04	0.49 – 2.20	0.923	0.96	0.51 – 1.83	0.904	0.95	0.33 – 2.76	0.932	2.36	1.00 – 5.54	**0.049**	1.09	0.66 – 1.81	0.726
OpennessToExperience Factor	0.74	0.34 – 1.60	0.449	1.19	0.62 – 2.31	0.602	1.70	0.78 – 3.68	0.180	1.21	0.65 – 2.25	0.552	1.52	0.53 – 4.35	0.431	0.89	0.40 – 1.96	0.769	1.04	0.64 – 1.70	0.861
Agreeableness Factor	1.42	0.57 – 3.54	0.448	0.99	0.45 – 2.19	0.987	0.85	0.33 – 2.16	0.725	0.48	0.22 – 1.01	0.052	0.37	0.09 – 1.44	0.150	0.71	0.29 – 1.73	0.455	0.72	0.40 – 1.29	0.271
Conscientiousness Factor	0.90	0.37 – 2.20	0.822	1.16	0.53 – 2.52	0.711	1.05	0.43 – 2.51	0.921	0.74	0.37 – 1.51	0.411	0.66	0.20 – 2.12	0.482	1.41	0.60 – 3.31	0.433	1.27	0.72 – 2.25	0.403
**Random Effects**																					
σ^2	3.29			3.29			3.29			3.29			3.29			3.29			3.29		
τ_{00}	6.52 $_{Participant\_ID}$			4.78 $_{Participant\_ID}$			4.04 $_{Participant\_ID}$			3.90 $_{Participant\_ID}$			8.00 $_{Participant\_ID}$			3.75 $_{Participant\_ID}$			2.33 $_{Participant\_ID}$		
	0.54 $_{Object\_ID}$			1.50 $_{Object\_ID}$			2.33 $_{Object\_ID}$			0.28 $_{Object\_ID}$									1.30 $_{Object\_ID}$		
ICC	0.68			0.66			0.66			0.56			0.71			0.53			0.52		
N	52 $_{Participant\_ID}$			52 $_{Participant\_ID}$			52 $_{Participant\_ID}$			52 $_{Participant\_ID}$			52 $_{Participant\_ID}$			52 $_{Participant\_ID}$			52 $_{Participant\_ID}$		
	132 $_{Object\_ID}$			132 $_{Object\_ID}$			132 $_{Object\_ID}$			132 $_{Object\_ID}$									132 $_{Object\_ID}$		
Observations	6864			6864			6864			6864			6864			6864			6864		
Marginal R^2 / Conditional R^2	0.048 / 0.697			0.016 / 0.661			0.096 / 0.692			0.150 / 0.626			0.116 / 0.742			0.139 / 0.598			0.025 / 0.537		
