# Supplementary material for: Emotional Daily Life Library (E-DLL): Validation of a database of 3D objects for emotion elicitation
Source: Int J Clin Health Psychol. 2026 May 14;26(2):100690. doi: 10.1016/j.ijchp.2026.100690 (PMC13202292; doi:10.1016/j.ijchp.2026.100690)
Supplement: MMC S4 [file mmc4.pdf]

Supplementary Material 4 - Spearman correlations between cognitive and perceptual dimensions (Bonferroni adjusted)

Parameter1	Parameter2	rho	CI	CI_low	CI_high	S	p	Method	n_Obs
Recognition	Name	0.6044707	0.95	0.4796237	0.7053300	151608.76	0.0000000	Spearman correlation	132
Recognition	Familiarity	0.7395618	0.95	0.6480008	0.8100513	99827.54	0.0000000	Spearman correlation	132
Recognition	Category	0.3072075	0.95	0.1388887	0.4582752	265551.52	0.0050946	Spearman correlation	132
Recognition	VisualComplexity	0.5066132	0.95	0.3631384	0.6266190	189118.12	0.0000000	Spearman correlation	132
Recognition	ObjectInteraction	0.3255071	0.95	0.1587732	0.4741895	258537.17	0.0020974	Spearman correlation	132
Name	Familiarity	0.4583323	0.95	0.3072730	0.5868612	207624.47	0.0000005	Spearman correlation	132
Name	Category	0.1401059	0.95	-0.0366168	0.3083313	329602.56	1.0000000	Spearman correlation	132
Name	VisualComplexity	0.4067130	0.95	0.2486747	0.5436575	227410.46	0.0000196	Spearman correlation	132
Name	ObjectInteraction	0.2594422	0.95	0.0876183	0.4162744	283860.24	0.0399648	Spearman correlation	132
Familiarity	Category	0.1384095	0.95	-0.0383443	0.3067650	330252.80	1.0000000	Spearman correlation	132
Familiarity	VisualComplexity	0.4495405	0.95	0.2972110	0.5795541	210994.41	0.0000010	Spearman correlation	132
Familiarity	ObjectInteraction	0.4997475	0.95	0.3551307	0.6210032	191749.77	0.0000000	Spearman correlation	132
Category	VisualComplexity	0.3072727	0.95	0.1389593	0.4583321	265526.52	0.0050791	Spearman correlation	132
Category	ObjectInteraction	0.0133525	0.95	-0.1628505	0.1887301	378187.91	1.0000000	Spearman correlation	132
VisualComplexity	ObjectInteraction	0.2180536	0.95	0.0439166	0.3793314	299724.74	0.1802175	Spearman correlation	132
